# Little composite dark matter

**DOI:** 10.1140/epjc/s10052-018-5552-3

**Published:** 2018-02-05

**Authors:** Reuven Balkin, Gilad Perez, Andreas Weiler

**Affiliations:** 1 First Physik-Department, Technische Universität München, 85748 Garching, Germany; 2 Department of Particle Physics and Astrophysics, Weizmann Institute of Science, Rehovot, 7610001 Israel

## Abstract

We examine the dark matter phenomenology of a composite electroweak singlet state. This singlet belongs to the Goldstone sector of a well-motivated extension of the Littlest Higgs with *T*-parity. A viable parameter space, consistent with the observed dark matter relic abundance as well as with the various collider, electroweak precision and dark matter direct detection experimental constraints is found for this scenario. *T*-parity implies a rich LHC phenomenology, which forms an interesting interplay between conventional natural SUSY type of signals involving third generation quarks and missing energy, from stop-like particle production and decay, and composite Higgs type of signals involving third generation quarks associated with Higgs and electroweak gauge boson, from vector-like top-partners production and decay. The composite features of the dark matter phenomenology allows the composite singlet to produce the correct relic abundance while interacting weakly with the Higgs via the usual Higgs portal coupling $\lambda _{\text {DM}}\sim O(1\%)$, thus evading direct detection.

## Introduction

The Hierarchy problem of the Standard Model (SM) could be solved by assuming that the Higgs is a pseudo Nambu-Goldstone Boson (pNGB) of a spontaneously broken global symmetry [1–4]. In this scenario, the Higgs is not an elementary particle but rather a composite state whose constituents are held together by some new strong force. In this respect, the Composite Higgs resembles other scalars found in nature, the QCD pions. An extended composite sector could also explain the origin of dark matter (DM) [5–12]. The same strong dynamics responsible for the Higgs may produce a stable neutral scalar bound state, a composite DM candidate. This could be considered in analogy to the Proton, another QCD bound state, which is an abundant particle in our universe, whose stability is insured by an (accidental) global symmetry. The composite DM candidate is a pNGB and it could be naturally as light as the weak scale, which fits in the weakly interacting massive particle (WIMP) paradigm.

One realization of the composite Higgs scenario is the Littlest Higgs [13–19]. The original model is strongly constrained by electroweak precision tests (EWPT) due to tree level contributions to electroweak observables [20–27]. These constraints required the symmetry breaking scale *f* to be a few TeV, thus reintroducing considerable fine-tuning.

*T*-Parity has been proposed in order to prevent tree-level exchanges of heavy states [28–31]. The new heavy states are odd under a discrete *T*-parity, therefore contributions to electroweak observables are possible only at the 1-loop level. This allows the symmetry breaking scale *f* to be *O*(1) TeV. As an added benefit, *T*-Parity can be used as a stabilizing symmetry for a DM candidate, as the lightest *T*-odd particle is guaranteed to be stable.

In this work, we consider the phenomenology of a Littlest Higgs model with *T*-parity (LHT) with a consistent implementation of *T*-parity in the fermionic sector [32]. Compared to the simplest LHT model, one enlarges the symmetry breaking pattern and also the unbroken symmetry group *H* which allows a complete composite representation containing just one fermion doublet. In particular, we analyze the DM phenomenology of a composite singlet scalar. In Sect. 2 we present the model and motivate the extension leading to the larger Goldstone sector. In Sect. 3 we briefly review the scalar potential structure. A detailed discussion of the scalar potential can be found in Appendix 1. In Sects. 4 and 5 we derive constraints on the model parameters from recent LHC searches and EWPT. In Sect. 6 we discuss the DM phenomenology of the composite singlet DM. We finally summarize our results and conclude in Sect. 7.

## Model

The model is based on the Littlest Higgs (LH) $\frac{SU(5)}{SO(5)}$ non-linear sigma model [13]. We define the scalar field $\Sigma$ in the symmetric $\mathbf {15}$ representation of *SU*(5):

1 $$\begin{aligned} \Sigma \rightarrow U \Sigma U^T. \end{aligned}$$

$\Sigma$ develops a vacuum expectation value (VEV),

2 $$\begin{aligned} \langle \Sigma \rangle \equiv \Sigma _0 = \begin{pmatrix} &{} &{} \mathbb {1}_2 \\ &{} 1 &{} \\ \mathbb {1}_2 &{} &{} \end{pmatrix}, \end{aligned}$$

spontaneously breaking *SU*(5) to *SO*(5). The 10 unbroken *SO*(5) generators denoted by $T_i$ satisfy

3 $$\begin{aligned} T_i\Sigma _0 + \Sigma _0 T^T_i = 0, \end{aligned}$$

and the 14 broken SU(5) generators denoted by $X_j$ satisfy

4 $$\begin{aligned} X_j\Sigma _0 - \Sigma _0 X^T_j = 0. \end{aligned}$$

We gauge two subgroups of *SU*(5), denoted by $[SU(2)~\times ~ U(1)]_i$ with $i=1,2$. The gauged generators are

5a $$\begin{aligned} Q_1^a\equiv & {} \begin{pmatrix} \frac{\sigma ^a}{2} &{} 0 &{} 0\\ 0&{} 0&{} 0 \\ 0&{} 0&{} 0 \\ \end{pmatrix}, \quad Y_1 =\frac{1}{10}\begin{pmatrix} 3 &{}&{}&{}&{} \\ &{}3&{}&{}&{} \\ &{}&{}-2&{}&{}\\ &{}&{}&{}-2&{}\\ &{}&{}&{}&{}-2\\ \end{pmatrix} , \end{aligned}$$

5b $$\begin{aligned} Q_2^a\equiv & {} \begin{pmatrix} 0 &{} 0 &{} 0\\ 0&{} 0&{} 0 \\ 0&{} 0&{} \frac{-{\sigma ^a}^*}{2} \\ \end{pmatrix}, \quad Y_2 =\frac{1}{10}\begin{pmatrix} 2 &{}&{}&{}&{} \\ &{}2&{}&{}&{} \\ &{}&{}2&{}&{}\\ &{}&{}&{}-3&{}\\ &{}&{}&{}&{}-3\\ \end{pmatrix} . \end{aligned}$$

$\sigma ^a$ with $a=1,2,3$ are the Pauli matrices.

$\Sigma _0$ spontaneously breaks the gauge symmetry to its diagonal subgroup $[SU(2)~\times ~U(1)]_{1+2}$, which we identify as the SM electroweak gauge group. The Nambu-Goldstone bosons (NGB’s) associated with the broken *SU*(5) generators decompose under the SM gauge group to the following representations

6 $$\begin{aligned} \mathbf {1}_0 \oplus \mathbf {3}_0 \oplus \mathbf {2}_{1/2} \oplus \mathbf {3}_{1}. \end{aligned}$$

We can parameterize the low energy degrees of freedom of the $\Sigma$ field using the NGB’s, defining $\Pi _\Sigma \equiv \pi _a X_a$:

7a $$\begin{aligned}&\Sigma = e^{i\Pi _\Sigma /f} \Sigma _0 e^{i\Pi ^T_\Sigma /f} = e^{2i\Pi _\Sigma /f} \Sigma _0,\end{aligned}$$

7b $$\begin{aligned}&\Pi _\Sigma = \begin{pmatrix} \frac{\tau \cdot \sigma }{2}+ \frac{\phi _0}{2 \sqrt{5}} \mathbb {1}_2 &{} \frac{H}{\sqrt{2}} &{} \Phi \\ \frac{H^\dagger }{\sqrt{2}} &{}- \frac{2\phi _0}{ \sqrt{5}} &{} \frac{H^T}{\sqrt{2}} \\ \Phi ^\dagger &{} \frac{H^*}{\sqrt{2}} &{} \frac{\tau \cdot \sigma ^*}{2}+ \frac{\phi _0}{2 \sqrt{5}} \mathbb {1}_2 \end{pmatrix},\quad \end{aligned}$$

7c $$\begin{aligned}&\text {with}\,\,\, \Phi = \begin{pmatrix} \Phi ^{++} &{} \Phi ^+ / \sqrt{2} \\ \Phi ^+ / \sqrt{2} &{} \Phi ^0 \end{pmatrix}. \end{aligned}$$

In the original LH model, the triplet $\tau$ and the singlet $\phi _0$ are “eaten” by the heavy gauge bosons. The physical scalar spectrum contains the complex doublet *H* which we identify as the SM Higgs field, and a heavy charged triplet $\Phi$. The gauge spectrum contains the SM gauge fields and additional heavy gauge fields with masses $m_{W_H}\sim gf, m_{B_H}\sim g' f,$ with $g,g'$ the SM gauge couplings. The heavy gauge states contribute at tree level to the electroweak oblique parameters. These contributions lead to stringent constraints from electroweak precision tests (EWPT), pushing the symmetry breaking scale of the original LH model $f \sim$ a few TeV (e.g Ref. [20]). The corrections to electroweak observables from the heavy gauge states are made smaller by introducing a discrete symmetry which forbids tree level exchanges of heavy states. The addition of a discrete symmetry stabilizes the lightest odd particle, making it a viable DM candidate. This discrete symmetry, usually referred to as *T*-parity, is defined as [28]

8 $$\begin{aligned} \text {{ T}-parity: }\,\, T_i \rightarrow \Omega T_i \Omega , \quad X_j \rightarrow -\Omega X_j\Omega \end{aligned}$$

with

9 $$\begin{aligned} \Omega = -\exp [2 \pi i Q^3_{1+2} ] = \text {diag}(1,1,-1,1,1), \end{aligned}$$

which is an automorphism defined on the *SU*(5) generators. This definition determines the *T*-parity of all the fields associated with the *SU*(5) generators, namely the Goldstone and gauge fields. The $\Omega$ rotation is introduced to make the Higgs even under *T*-parity, while keeping the rest of the Goldstone fields odd. For the gauge fields, the *T*-parity transformation can be interpreted as an exchange symmetry between the gauge groups $1 \leftrightarrow 2$. Hence the diagonal combination is even, and the broken combination is odd.

Let us understand how linear representations of *SU*(5) transform under *T*-parity. One can use Eq. (9) to show that each transformation $g = e^{i \alpha _j X_j + i \beta _i T_i}\in SU(5)$ is mapped under *T*-parity to

10 $$\begin{aligned} g ~\rightarrow ~ \tilde{g} \equiv \Omega \Sigma _0 g^* \Sigma _0 \Omega . \end{aligned}$$

Therefore, up to a constant matrix, fundamental and anti-fundamental indices of *SU*(5) are mapped to each other

11 $$\begin{aligned} \underbrace{V_i}_{\mathbf {5}} ~\leftrightarrow ~ (\Sigma _0 \Omega )_{ij}\underbrace{U^j}_{\overline{\mathbf {5}}}. \end{aligned}$$

The $\Sigma$ field transforms with two fundamental *SU*(5) indices, so under *T*-parity

12 $$\begin{aligned} \Sigma ~\rightarrow ~\tilde{\Sigma }\equiv \Omega \Sigma _0 \Sigma ^\dagger \Sigma _0 \Omega . \end{aligned}$$

### A UV doubling problem, making the *T*-odd doublet massive

The coset structure of LH with *T*-parity is in tension with the SM matter content [32, 33]. The low energy theory must contain a *T*-even massless *SU*(2) doublet, the left-handed quark doublet of the SM. Since *T*-parity can be understood as an exchange symmetry between the two gauged *SU*(2) subgroups of *SU*(5) (we omit the *U*(1) factors for the following discussion), one must therefore introduce two doublets $\psi _i$, each transforming under a different $SU(2)_i$ with $i=1,2$. Under *T*-parity the two doublets are mapped into each other

13 $$\begin{aligned} \psi _1 ~\leftrightarrow ~ \psi _2. \end{aligned}$$

We would like to write a mass term for the *T*-odd combination $\psi _- \equiv (\psi _1-\psi _2)$ that respects the SM gauge group. Let us introduce a right-handed field $\psi ^c$ transforming as a doublet under the SM gauge group $[SU(2)]_{1+2}$

14 $$\begin{aligned} \mathcal {L}\ni (\overline{\psi }_1-\overline{\psi }_2)\psi ^c. \end{aligned}$$

This term respects the SM gauge group, however each term by itself breaks $SU(2)_1 ~\times ~ SU(2)_2$ and cannot be generated by a reasonable UV theory which respects those gauge symmetries, unless they are spontaneously broken. Assuming that $\psi ^c$ cannot be a doublet of just one of the $SU(2)'s$, we expect the mass term to arise as a result of spontaneous symmetry breaking

15 $$\begin{aligned} \mathcal {L}\ni (\overline{\psi }_1\langle \phi _1\rangle -\overline{\psi }_2\langle \phi _2\rangle )\psi ^c, \end{aligned}$$

where we introduced two sources of spontaneous symmetry breaking, the VEV’s $\langle \phi _1\rangle$ and $\langle \phi _2\rangle$.

Let us examine now the VEV’s which we can use to write this term in a gauge-invariant way. In Sects. 2.1.1 and 2.1.2 we briefly examine two different constructions presented in the literature that generate the mass term of Eq. (14). We mention possible shortcomings of these constructions, which motivate the construction used in this work, presented in Sect. 2.1.3. Readers interested only in the details of the model used in this work, may skip directly to Sect. 2.1.3.

#### Non-linear formulation of a massive odd doublet

One construction commonly presented in the literature uses the CCWZ formalism [34, 35]. The main advantage of this approach is that no new sources of spontaneous symmetry breaking are needed.

First we have the linear representations of *SU*(5) [29]

16 $$\begin{aligned} \Psi _1 = \begin{pmatrix} \psi _1 \\ 0 \\ 0 \end{pmatrix}_{\overline{\mathbf {5}}}, \quad \Psi _2 = \begin{pmatrix} 0 \\ 0 \\ \psi _2 \end{pmatrix}_{\mathbf {5}}, \end{aligned}$$

with the following *T*-parity transformation

17 $$\begin{aligned} \Psi _1 ~\rightarrow ~ \Omega \Sigma _0 \Psi _2. \end{aligned}$$

A mass term for the *T*-odd combination is constructed using a non-linearly transforming field

18 $$\begin{aligned} \tilde{\Psi }^c = \begin{pmatrix} \psi ^c_1 \\ \chi ^c \\ \psi ^c_2 \end{pmatrix},\quad \text { a }{\mathbf {5}} \text { of } SO(5). \end{aligned}$$

Under a transformation $$g\in SU(5)$$

19 $$\begin{aligned} \tilde{\Psi }^c ~\rightarrow ~ O(\Pi _\Sigma ,g) \tilde{\Psi }^c, \quad O \in SO(5). \end{aligned}$$

$e^{i\Pi _\Sigma /f}$ transforms under a transformation $g\in SU(5)$ in the following way

20 $$\begin{aligned} e^{i\Pi _\Sigma /f} ~\rightarrow ~ g e^{i\Pi _\Sigma /f} O^\dagger = O e^{i\Pi _\Sigma /f} (\Sigma _0 g^T \Sigma _0). \end{aligned}$$

The kinetic term for $\tilde{\Psi }^c$ contains the $e_\mu$ symbol defined by [34, 35]

21 $$\begin{aligned} ie^{-i\Pi _\Sigma /f} (\partial _\mu e^{i\Pi _\Sigma /f}) \equiv d^j_\mu X^{j}+e^i_\mu T^{i}. \end{aligned}$$

Using the automorphism defined in Eq. (9) we can write $e_\mu \equiv e^i_\mu T^{i}$ in a *T*-parity symmetric form

22 $$\begin{aligned} e_\mu = \frac{i}{2} \left( e^{-i\Pi _\Sigma /f} \partial _\mu e^{i\Pi _\Sigma /f} + e^{i\Pi _\Sigma /f} \partial _\mu e^{-i\Pi _\Sigma /f}\right) . \end{aligned}$$

The $e_\mu$ symbol transform as a covariant derivative

23 $$\begin{aligned} (\partial _\mu +e_\mu )~\rightarrow ~ O(\partial _\mu +e_\mu )O^\dagger , \end{aligned}$$

which allows us to write an invariant kinetic term for $\tilde{\Psi }^c$. Note that under *T*-parity

24 $$\begin{aligned} e_\mu \rightarrow \Omega e_\mu \Omega , \end{aligned}$$

therefore the transformation of $\tilde{\Psi }^c$ under *T*-parity is

25 $$\begin{aligned} \tilde{\Psi }^c \rightarrow -\Omega \tilde{\Psi }^c. \end{aligned}$$

The benefit of the CCWZ formalism is that the pion matrix can be used to “dress” the field $\tilde{\Psi }^c$ as linear representations of *SU*(5), e.g ${\mathbf {5}}$ and ${\bar{\mathbf {5}}}$

26 $$\begin{aligned} e^{i\Pi _\Sigma /f}\tilde{\Psi }^c ~\rightarrow ~ g(e^{i\Pi _\Sigma /f}\tilde{\Psi }^c), \end{aligned}$$

and

27 $$\begin{aligned} \,\,\Sigma _0e^{-i\Pi _\Sigma /f}\tilde{\Psi }^c ~\rightarrow ~ g^*(\Sigma _0e^{-i\Pi _\Sigma /f}\tilde{\Psi }^c), \end{aligned}$$

with $g\in SU(5)$. Finally the mass term is given by [29]28The field $\tilde{\Psi }^c$ must be a complete *SO*(5) representation, otherwise the kinetic term for $\tilde{\Psi }^c$ would explicitly break the global symmetry protecting the Higgs mass [29]. The field $\psi _1^c$ is still massless at this point. One could formally introduce an additional doublet $\eta$ and write a mass term

29 $$\begin{aligned} \mathcal {L}\ni M (\bar{\eta }\psi _1^c + \text {h.c}). \end{aligned}$$

This term breaks the global symmetries protecting the Higgs mass, generating $O(M^2)$ contributions to the Higgs mass.

#### Adding a third $SU(2)~\times ~ U(1)$

We conclude that the model requires additional structure in order to give mass to the *T*-odd combination without explicit breaking of the global symmetry.

One possible solution is to add an additional gauge group [33, 36], denoted by $[SU(2)~\times ~U(1)]_3$. Now $\psi ^c$ of Eq. (15) transforms as a doublet under $[SU(2)~\times ~ U(1)]_3$ and the scalars $\phi _i$ transform as a bi-fundamentals of $[SU(2)\times U(1)]_i ~\times ~ [SU(2)~\times ~ U(1)]_3$ with $i=1,2$. This solution introduces new heavy *T*-even gauge fields. The new *T*-even gauge fields can be made heavy by making the coupling constant of the third $SU(2)~\times ~U(1)$ gauge group large, effectively decoupling them from the theory without spoiling the naturalness of the model. One has the choice of how to enlarge the global symmetry to incorporate this additional gauge group. The most naive extension is

30 $$\begin{aligned} SU(5) \rightarrow SU(5) ~\times ~ [SU(2) ~\times ~ U(1)]_3 \end{aligned}$$

We introduce additional scalars $\Phi _1$ and $\Phi _2$ transform under the enlarged group as $({\bar{\mathbf {5}}},{\bar{\mathbf {2}}})$ and $({\mathbf {5}},{\bar{\mathbf {2}}})$ respectively (disregarding the *U*(1) charges), namely

31 $$\begin{aligned} \Phi _1\rightarrow & {} g^* \Phi _1 g_3^\dagger , \quad \Phi _2 \rightarrow g \Phi _2 g_3^\dagger , \nonumber \\ g\in & {} SU(5),\quad g_3 \in [SU(2)~\times ~ U(1)]_3. \end{aligned}$$

Under *T*-parity

32 $$\begin{aligned} \Phi _1 \rightarrow \Sigma _0 \Omega \Phi _2, \quad \psi ^c \rightarrow -\psi ^c. \end{aligned}$$

The *T*-odd doublet gets a mass33after $\Phi _1$ and $\Phi _2$ acquire VEV’s given by

34 $$\begin{aligned} \langle \Phi _1\rangle = f\begin{pmatrix} \mathbb {1}_{2~\times ~2} \\ 0_{1~\times ~2}\\ 0_{2~\times ~2}\\ \end{pmatrix} = \Sigma _0 \Omega \langle \Phi _2\rangle . \end{aligned}$$

The appearance of $\Phi _1,\Phi _2$ results in a deviation from the original coset structure of the LH, with the altered coset structure

35 $$\begin{aligned} \frac{SU(5) ~\times ~ SU(2) ~\times ~ U(1)}{[SU(2) ~\times ~ U(1)]_{1+2+3}}. \end{aligned}$$

We now identify $[SU(2) ~\times ~ U(1)]_{1+2+3}$ as the SM gauge group. This coset contains in the original 14 NGB’s of the LH coset, and additional 10 NGB’s from the spontaneously broken *SO*(5). These 10 additional states decompose under the SM gauge group as

36 $$\begin{aligned} \mathbf {1}_0 \oplus \mathbf {3}_0 \oplus \mathbf {2}_{1/2} \oplus \mathbf {1}_{1/2}. \end{aligned}$$

The additional neutral singlet $\mathbf {1}_0$ and triplet $\mathbf {3}_0$ are “eaten” by the additional *T*-even gauge fields. This naive approach unavoidably introduces additional physical NGB’s in the form of a *T*-odd doublet $\mathbf {2}_{1/2}$ and a *T*-even complex scalar $\mathbf {1}_{1/2}$. These states must be made massive without spoiling the symmetry protection of the SM Higgs. Additional NGB’s are a generic result of the enlarged global symmetry structure, even more so when the additional *SU*(2) is a gauged subgroup of a larger global symmetry [33].

#### Mirroring the $1 \leftrightarrow 2$ exchange symmetry

In this work we consider a concrete solution suggested in Ref. [32]. We extend the global symmetry

37 $$\begin{aligned} SU(5) \!\rightarrow \! SU(5) \times [SU(2)\times U(1)]_L \times [SU(2)\times U(1)]_R.\nonumber \\ \end{aligned}$$

We introduce a scalar field, *X*, which transforms linearly under $[SU(2)\times U(1)]_L \times [SU(2)\times U(1)]_R$

38 $$\begin{aligned} X \rightarrow g_L X g_R^{\dagger }. \end{aligned}$$

When the $\Sigma$ and *X* acquire VEV’s, $\langle \Sigma \rangle = \Sigma _0$ and $\langle X \rangle = \mathbb {1}_2$, the symmetry is spontaneously broken to

39 $$\begin{aligned} \frac{SU(5)}{SO(5)} \times \frac{[SU(2)\times U(1)]_L \times [SU(2)\times U(1)]_R}{[SU(2)\times ~U(1)]_V}. \end{aligned}$$

We gauge two $SU(2)~\times ~ U(1)$ subgroups defined as the combinations $[SU(2)~\times ~ U(1)]_{1+L}$ and $[SU(2)~\times ~ U(1)]_{2+R}$. The residual gauge symmetry $[SU(2)~\times ~ U(1)]_{1+2+L+R}$ is identified as the SM gauge group. We can parametrise *X* using the non-linearly transforming Goldstone fields associated with this symmetry breaking,

40a $$\begin{aligned} X\equiv & {} e^{\frac{i}{f'}\Pi _X}\langle X \rangle e^{\frac{i}{f'}\Pi _X} = e^{\frac{2i}{f'}\Pi _X},\end{aligned}$$

40b $$\begin{aligned} \Pi _X= & {} \frac{1}{2} \left( \pi _i \sigma ^i + \pi _0 \mathbb {1}_2 \right) . \end{aligned}$$

Note that the symmetry breaking scale $f'$ may be different than *f*, the symmetry breaking scale of the original coset defined in Eq. (7). *T*-parity in the additional coset is realized as an $L~\leftrightarrow ~R$ exchange, mirroring the $1 \leftrightarrow 2$ exchange symmetry of the original coset. Under *T*-parity,

41 $$\begin{aligned} \Pi _X \rightarrow -\Pi _X, \end{aligned}$$

We introduce a non-linear representation of

$$\begin{aligned} \,[SU(2)\times U(1)]_L\times [SU(2) \times U(1)]_R. \end{aligned}$$

$\psi ^c$ is a doublet of the unbroken subgroup $[SU(2)\times U(1)]_{L+R}$, transforming non-linearly under

$$\begin{aligned} g_L,g_R \in [SU(2)\times U(1)]_L\times [SU(2)\times U(1)]_R, \end{aligned}$$

42a $$\begin{aligned}&\psi ^c \rightarrow V(\Pi _X,g_L,g_R)\psi ^c, \end{aligned}$$

42b $$\begin{aligned}&V \in [SU(2)\times U(1)]_{L+R}. \end{aligned}$$

The transformation properties under $[SU(2)\times U(1)]_L\times [SU(2)\times U(1)]_R \nonumber$ of $e^{i \Pi _X/f'}$ in this case are

43 $$\begin{aligned} e^{i \Pi _X/f'} \rightarrow g_L e^{i \Pi _X/f'} V^\dagger = V e^{i \Pi _X/f'} g_R^\dagger . \end{aligned}$$

This object can be used to “dress” $\psi ^c$ as linear representations

44a $$\begin{aligned}&e^{i \Pi _X/f'}\psi ^c \rightarrow g_L (e^{i \Pi _X/f'}\psi ^c),\end{aligned}$$

44b $$\begin{aligned}&e^{-i \Pi _X/f'}\psi ^c \rightarrow g_R (e^{i \Pi _X/f'}\psi ^c). \end{aligned}$$

Finally the mass term can be written as [32]

45 $$\begin{aligned} \mathcal {L}\ni (\overline{\psi }_1 e^{i\Pi _X/f'}-\overline{\psi }_2 \Sigma _0 e^{-i\Pi _X/f'})\psi ^c+\text {h.c}. \end{aligned}$$

This extension allows us to add a single *SU*(2) doublet to the spectrum, $\psi ^c$, and write a mass term for the *T*-odd doublet, without any explicit breaking of the global symmetry. In additional to the 14 original NGB’s of Eq. (6), our spectrum includes now an additional NGB’s, a real singlet $\mathbf {1}_0$ and a real triplet $\mathbf {3}_0$.

### Gauge sector

We write the Lagrangian for the non-linear $\sigma$ model

46 $$\begin{aligned} \mathcal {L}_{nl\sigma }= & {} \frac{f^2}{8}\text {Tr}[(D_\mu \Sigma )(D^\mu \Sigma ^*)] + \frac{f'}{4}\text {Tr}[(D_\mu X )(D^\mu X^\dagger ) ]. \nonumber \\ \end{aligned}$$

We parameterize $\Sigma ,X$ using the NGB’s as defined in Eq. (7) and Eq. (40). The exact form of the covariant derivatives can be found in App. Appendix A

Once we set $\Sigma ,X$ to their respected VEV’s, we find that the following linear combinations,

47 $$\begin{aligned} W^a_H = \frac{1}{\sqrt{2}}(W_1^a-W_2^a),\quad B_H = \frac{1}{\sqrt{2}}(B_1-B_2), \end{aligned}$$

acquire a mass

48 $$\begin{aligned} M^2_{W_H} = g^2 f^2 (1+r^2), M^2_{B_H} = \frac{1}{5} {g'}^2 f^2 \left( 1+\frac{1}{5}{r}^2\right) , \end{aligned}$$

with

49 $$\begin{aligned} r\equiv \frac{f'}{f}. \end{aligned}$$

We recognize the orthogonal linear combinations,

50 $$\begin{aligned} W^a = \frac{1}{\sqrt{2}}(W_1^a+W_2^a),\quad B = \frac{1}{\sqrt{2}}(B_1+B_2), \end{aligned}$$

as the SM gauge fields.

### Goldstone sector

In addition to the complex Higgs doublet *H* and the charged triplet $\Phi$, the Goldstone sector includes additional physical states: a real singlet *s* and a real triplet $\varphi \equiv \frac{1}{2} \varphi _a \sigma ^a$, defined as the following linear combinations

51 $$\begin{aligned} s = c_0 \pi _0+s_0 \phi _0,\quad \varphi _a = c_{3} \tau _a-s_{3} \pi _a, \end{aligned}$$

with the mixing angles

52a $$\begin{aligned} s_0= & {} \sqrt{1-c_0^2} \equiv \frac{r}{\sqrt{5+r^2}},\end{aligned}$$

52b $$\begin{aligned} c_3= & {} \sqrt{1-s_3^2} \equiv \frac{r}{\sqrt{1+r^2}}. \end{aligned}$$

The orthogonal linear combinations,

53 $$\begin{aligned} G_0 = -s_0 \pi _0+c_0 \phi _0,\quad G^a = s_{3} \tau _a+c_{3} \pi _a, \end{aligned}$$

are “eaten” by the heavy gauge fields and removed from the spectrum in the unitary gauge.

### Matter sector

The top Yukawa generates the largest quadratically divergent contribution to the Higgs mass, therefore we limit our discussion to the third quark family. The terms in the top sector must respect enough of the global symmetries in order for the Higgs mass to be protected from 1-loop quadratically divergent contributions. This mechanism is usually referred to as “collective” symmetry breaking. In order to respect these symmetries we enlarge the multiplets introduced in Eq. (16) and introduce top partners. The quadratically divergent contribution to the Higgs mass from these top partners would eventually cancel out with the top contribution. We start by introducing left-handed Weyl fermions. We embed the doublets $\psi _{1,2}$ with the singlets $\chi _{1,2}$ (the top partners) in incomplete *SU*(5) multiplets

54 $$\begin{aligned} \Psi _1 = \begin{pmatrix} \psi _1 \\ \chi _1 \\ 0 \end{pmatrix}_{\overline{\mathbf {5}}}, \quad \Psi _2 = \begin{pmatrix} 0 \\ \chi _2 \\ \psi _2 \end{pmatrix}_{\mathbf {5}}. \end{aligned}$$

Under *T*-parity,

55 $$\begin{aligned} \Psi _1 \rightarrow \Omega \Sigma _0 \Psi _2, \end{aligned}$$

or equivalently

56 $$\begin{aligned} \psi _1 \leftrightarrow \psi _2, \quad \chi _1 \leftrightarrow -\chi _2. \end{aligned}$$

We introduce 3 right-handed singlets denoted by $\tilde{t}_R, \tau _{1,2}$. Under *T*-parity,

57 $$\begin{aligned} \tilde{t}_R \leftrightarrow \tilde{t}_R, \quad \tau _1 \leftrightarrow \tau _2. \end{aligned}$$

The top Yukawa is given by [13, 30]

58a $$\begin{aligned} \mathcal {L}_{\text {top}}= & {} \frac{\lambda _1 f}{2}\left( {\overline{\Psi }_1}_i O_i+ (\overline{\Psi }_2 \Omega \Sigma _0)_i \tilde{O}_i \right) \tilde{t}_R\end{aligned}$$

58b $$\begin{aligned}&+\frac{\lambda _2 f}{\sqrt{2}}\left( \overline{\chi }_1 \tau _1 - \overline{\chi }_2 \tau _2 \right) +\text {h.c}, \nonumber \\ O_i\equiv & {} \epsilon _{ijk}\Sigma _{j4}\Sigma _{k5},\quad \tilde{O}_i \equiv \epsilon _{ijk}\tilde{\Sigma }_{j4}\tilde{\Sigma }_{k5}. \end{aligned}$$

$\tilde{\Sigma }$ is defined in Eq. (12). The indices *i*, *j*, *k* are summed over 1, 2, 3. We define the *T*-parity eigenstates

59a $$\begin{aligned} \Psi _+= & {} \frac{1}{\sqrt{2}}\left( \Psi _1 +\Omega \Sigma _0 \Psi _2 \right) \equiv \begin{pmatrix} \sigma _2 Q_L \\ \chi _+ \\ 0 \end{pmatrix},\end{aligned}$$

59b $$\begin{aligned} \Psi _-= & {} \frac{1}{\sqrt{2}}\left( \Psi _1 -\Omega \Sigma _0 \Psi _2 \right) \equiv \begin{pmatrix} \sigma _2 \psi ^-_L \\ T^-_L \\ 0 \end{pmatrix}, \end{aligned}$$

with

60a $$\begin{aligned} Q_L= & {} \begin{pmatrix} \tilde{t}_L \\ b_L \end{pmatrix} = \frac{1}{\sqrt{2}}\sigma _2(\psi _1+\psi _2), \end{aligned}$$

60b $$\begin{aligned} \psi ^-_L= & {} \frac{1}{\sqrt{2}}\sigma _2(\psi _1-\psi _2). \end{aligned}$$

The singlet *T*-parity eigenstates are defined as

61a $$\begin{aligned} \chi _+= & {} \frac{1}{\sqrt{2}}\left( \chi _1 - \chi _2 \right) , \quad \tau _+ = \frac{1}{\sqrt{2}}\left( \tau _1 + \tau _2 \right) , \end{aligned}$$

61b $$\begin{aligned} T^-_L= & {} \frac{1}{\sqrt{2}}\left( \chi _1 + \chi _2 \right) , \quad T^-_R = \frac{1}{\sqrt{2}}\left( \tau _1 - \tau _2 \right) . \end{aligned}$$

Note that the *T*-even fields, and in particular $\tilde{t}_L,\tilde{t}_R$, are not the mass eigenstates (hence the tilde). After the Higgs field acquires its VEV, $\langle H \rangle = \frac{1}{\sqrt{2}}( 0,v)^T$, we find the following mass matrix for the *T*-even fermions

62 $$\begin{aligned} \mathcal {L}_{\text {top}}\ni f\begin{pmatrix} \overline{\tilde{t}}_L&\overline{\chi }_+ \end{pmatrix} \begin{pmatrix} \frac{\lambda _1 s_v }{2}&{}0\\ \frac{\lambda _1 (1+c_v) }{2\sqrt{2}}&{} \frac{\lambda _2 }{\sqrt{2}} \end{pmatrix} \begin{pmatrix} \tilde{t}_R\\ \tau _+ \end{pmatrix}+\text {h.c}. \end{aligned}$$

We denoted

63 $$\begin{aligned} s_v = \sin \sqrt{2\xi }, \quad c_v = \cos \sqrt{2\xi }, \quad \xi \equiv \frac{v^2}{f^2}. \end{aligned}$$

The physical basis is given by

64a $$\begin{aligned} \begin{pmatrix} t_L \\ T^+_L \end{pmatrix} = \begin{pmatrix} c_L &{} -s_L \\ s_L &{}c_L \end{pmatrix} \begin{pmatrix} \tilde{t}_L \\ \chi _+ \end{pmatrix}, \end{aligned}$$

64b $$\begin{aligned} \begin{pmatrix} t_R \\ T^+_R \end{pmatrix} = \begin{pmatrix} c_R &{} -s_R \\ s_R &{} c_R \end{pmatrix} \begin{pmatrix} \tilde{t}_R \\ \tau _+ \end{pmatrix}, \end{aligned}$$

with $\sin \theta _{L/R} \equiv s_{L/R}$ and $\cos \theta _{L/R} \equiv c_{L/R}$. The mixing angles are given by [31]

65a $$\begin{aligned} \theta _L= & {} \frac{1}{2} \tan ^{-1} \left( \frac{2\sqrt{2} \lambda _1^2 s_v (1+c_v)}{4\lambda _2^2+(1+c_v)^2\lambda _1^2-2\lambda _1^2 s_v}\right) , \end{aligned}$$

65b $$\begin{aligned} \theta _R= & {} \frac{1}{2} \tan ^{-1} \left( \frac{4 \lambda _1 \lambda _2 (1+c_v)}{4\lambda _2^2-\lambda _1^2(2s_v^2+(1+c_v)^2)}\right) . \end{aligned}$$

The masses at leading order in $\xi$ are

66a $$\begin{aligned}&m_t^2 = \frac{1}{\sqrt{2}} \left( \frac{\lambda _1\lambda _2}{\sqrt{\lambda _1^2+\lambda _2^2}}\right) \sqrt{\xi } f, \end{aligned}$$

66b $$\begin{aligned}&m_{T^+} = \frac{\sqrt{\lambda _1^2+\lambda _2^2}}{\sqrt{2}}f. \end{aligned}$$

The top Yukawa coupling at leading order in $\xi$ is therefore

67 $$\begin{aligned} y_t = \frac{\lambda _1\lambda _2}{\sqrt{\lambda _1^2+\lambda _2^2}}. \end{aligned}$$

We shall keep $\lambda _2$ as a free parameter and fix $\lambda _1$ to produce the correct top Yukawa $y_t \approx 1$. The mixing angles at leading order in $\xi$ are

68a $$\begin{aligned}&s_L = \frac{\lambda _1^2}{\lambda _1^2+\lambda _2^2}\sqrt{\xi } = \left( \frac{y_t}{\lambda _2}\right) ^2\sqrt{\xi }, \end{aligned}$$

68b $$\begin{aligned}&s_R = \frac{\lambda _1}{\sqrt{\lambda _1^2+\lambda _2^2}} = \frac{y_t}{\lambda _2}. \end{aligned}$$

For the *T*-odd sector we must introduce a mass term for the doublet similar to the term in Eq. (45). We introduce a RH doublet $\psi _R^-$ transforming non-linearly under $[SU(2)~\times ~ U(1)]_L ~\times ~ [SU(2)~\times ~ U(1)]_R$ according to the CCWZ formalism. $\psi _R^-$ is odd under *T*-parity

69 $$\begin{aligned} \psi _R^- \rightarrow -\psi _R^-. \end{aligned}$$

The mass term is given by [32]

70 $$\begin{aligned} \mathcal {L}_{\kappa } = \frac{\kappa f}{\sqrt{2}}\left( \overline{\psi }_1 \sigma _2 e^{\frac{i}{f'}\Pi _X} - \overline{\psi }_2 \sigma _2 e^{-\frac{i}{f'}\Pi _X} \right) \psi _R^- +\text {h.c}. \end{aligned}$$

Our spectrum contains a *T*-odd singlet $T^-$ and a *T*-odd doublet $\psi ^-$ with the following masses

71 $$\begin{aligned} m_{T^-} = \frac{\lambda _2}{\sqrt{2}} f, \quad m_{\psi ^-} =\kappa f. \end{aligned}$$

Lastly, the explicit form of the kinetic terms can be found in App. Appendix A.

## Scalar potential

At tree level, the pNBG’s interact only through derivative interactions and their classical potential vanishes. The gauge and top sector couplings explicitly break the global symmetry. The classical scalar potential is radiatively generated from fermion and gauge loops. At 1-loop the fermion and gauge loops contributions are given by [37]

72a $$\begin{aligned}&V_{f}(H,\Phi ,s,\varphi ) = - \frac{N_c}{8 \pi ^2} \Lambda ^2a_1\,\text {Tr}\left[ M_{f}M^\dagger _{f} \right] \nonumber \\&\quad - \frac{N_c}{16 \pi ^2}a_2\text {Tr}\left[ \left( M_{f}M^\dagger _{f}\right) ^2 \log \left( \frac{M_{f}M^\dagger _{f} }{\Lambda ^2 } \right) \right] , \end{aligned}$$

72b $$\begin{aligned}&V_{\text {V}} (H,\Phi ,s,\varphi ) =\frac{3}{32 \pi ^2} \Lambda ^2 a_3\,\text {Tr}\left[ M^2_{\text {V}} \right] \nonumber \\&\quad + \frac{3}{64 \pi ^2}a_4\text {Tr}\left[ M^4_{\text {V}} \log \left( \frac{M^2_{\text {V}} }{\Lambda ^2 }\right) \right] , \end{aligned}$$

respectively. $M_{f}(H,\Phi ,s,\varphi )$ and $M^2_{\text {V}}(H,\Phi ,s,\varphi )$ are the fermion and gauge bosons mass matrices in the background of the pNGB’s. The $a_i$ parameters with $i=1,..,4$ are unknown *O*(1) numbers originating from unknown UV contributions to these operators. $\Lambda \sim 4 \pi f$ is the cutoff scale of the theory. Expanding the scalar potential $V = V_{f} +V_{\text {V}}$ in the NGB fields, we find that

73 $$\begin{aligned} V= & {} m_{\Phi }^2 \text {Tr}[\Phi ^\dagger \Phi ] -\mu ^2 |H|^2 + {m}_\varphi ^2 \text {Tr}[\varphi ^2]+\lambda |H|^4 \nonumber \\&+ \lambda _{\text {DM}}s^2 |H|^2 + \lambda _{\varphi }s H^\dagger \varphi H+.... \end{aligned}$$

We have omitted additional radiatively generated operators that are inconsequential for the upcoming discussions. A detailed analysis of the symmetries of the scalar potential of this model can be found in Appendix 1. In this section we summarize the most important features of the scalar potential.

The mass of the charged triplet $\Phi$ is quadratically divergent,

74 $$\begin{aligned} m_{\Phi } \sim \text {a few TeV}. \end{aligned}$$

We consider energy scales well below $m_{\Phi }$. We remove $\Phi$ from our spectrum by integrating it out. Due to *T*-parity, integrating out $\Phi$ at tree-level does not influence any of the couplings explicitly written in the scalar potential of Eq. (73). Like $m_{\Phi }^2$, the Higgs quartic $\lambda$ is generated by 1-loop quadratically divergent diagrams.

The rest of the operators in Eq. (73), including the Higgs mass term $\mu ^2$, are generated through logarithmically divergent loops, and as such they exhibit a mild dependence on the UV cutoff scale. The explicit calculations, found in Appendix 1, give us an order of magnitude estimation for the IR contribution to these operators at 1-loop. However quadratically divergent 2-loop diagrams as well as UV contributions can have comparable effects on these operators. Therefore we do not presume to be able to predict these couplings accurately in terms of the fundamental parameters of this model. In this work we treat the couplings in Eq. (73) as free parameters, except $\mu ^2$ and $\lambda$ which are already fixed by experiment. Our goal is to allow the free parameters to take values that are reasonable in light of the approximation given by the 1-loop IR contribution, and state explicitly when this is not the case.

In addition to ${m}_\varphi ^2,\lambda _{\text {DM}} ,\lambda _{\varphi }$, we must introduce a mass term for the singlet *s*. The singlet remains massless at 1-loop, and a mass for *s* is generated at the 2-loop level. We take the pre-EWSB mass term of the singlet, denoted as $\tilde{m}_s^2$, as a free parameter as well. The sizes and ranges of ${m}_\varphi ^2,\lambda _{\varphi },\tilde{m}_s^2,\lambda _{\text {DM}}$ are dictated by the DM phenomenology and are discussed in Sect. 6.

## LHC phenomenology

### *T*-even singlet $T^+$

The *T*-even singlet is responsible for cancelling the quadratically divergent top loop contribution to the Higgs mass, hence it is the standard top partner predicted by composite Higgs models. It can be doubly produced at the LHC via QCD processes, as well as singly produced with an associated third generation quark through the following EW interactions75In this model,

76a $$\begin{aligned} C_{bW}= & {} \sqrt{2} s_L \approx \frac{\sqrt{2 \xi }}{\lambda _2^2} \approx 0.35\left( \frac{1}{\lambda _2}\right) ^2 \left( \frac{1~\text {TeV}}{f}\right) , \end{aligned}$$

76b $$\begin{aligned} C_{tZ}= & {} \frac{ s_L c_L }{ c_W } \approx \frac{\sqrt{ \xi }}{ c_W \lambda _2^2} \approx 0.28\left( \frac{1}{\lambda _2}\right) ^2 \left( \frac{1~\text {TeV}}{f}\right) . \end{aligned}$$

#### Decay modes

We consider the limit $m_{T^+} \gg m_H,m_W,m_Z$. In this regime EWSB effects are negligible and we can formally take $\xi \rightarrow 0$. The dominant decays of $T^+$ are to the physical Higgs or to the longitudinal components of the SM gauge bosons with an associated third generation quark, in accordance with the equivalence theorem. We can parameterize the Higgs field in a general $R_\xi$ gauge using these would-be longitudinal components as

77 $$\begin{aligned} H = \begin{pmatrix} \phi ^+ \\ \frac{1}{\sqrt{2}}(v+ h+i \phi _0) \end{pmatrix}. \end{aligned}$$

The relevant interactions between the Higgs doublet and $T^+$ are

78 $$\begin{aligned} \mathcal {L}_{\text {top}}\ni&-\frac{1}{\sqrt{2}}\lambda _1s_R \left( \overline{t}_L(v+ h+i \phi _0)-\sqrt{2}\overline{b}_L\phi ^+\right) T^+_R, \end{aligned}$$

predicting that in the high energy limit,

79 $$\begin{aligned}&\text {Br}[T_+ \rightarrow h\; t] : \text {Br}[T_+ \rightarrow Z\; t] : \text {Br}[T_+ \rightarrow W^+\;b]\nonumber \\&\quad = 1 : 1 : 2. \end{aligned}$$

**Fig. 1:**
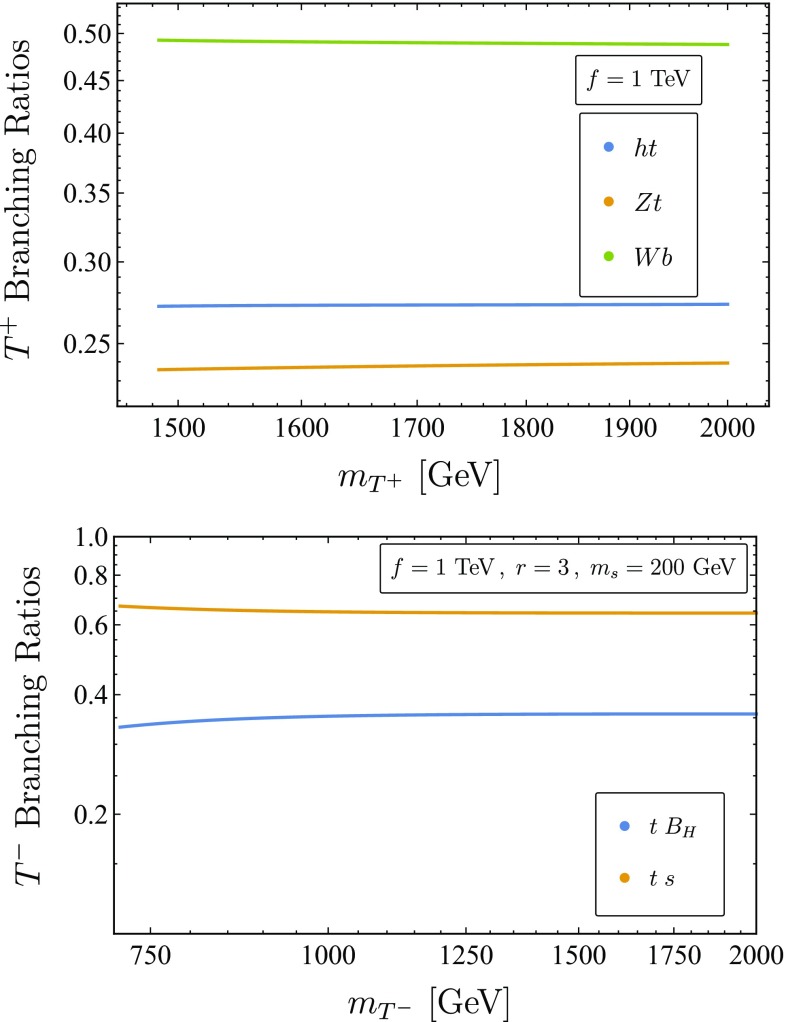
Left panel : Numeric results for branching ratios of $T^+$ for $f=1$ TeV. Right Panel: Numeric results for branching ratios of $T^-$ for $f=1$ TeV, $r=3$ and $m_s=200$ GeV. The mass of $B_H$ is a function of *f*, *r*, in this case $m_{B_H} = 270~$GeV

The exact branching ratios $T^{+}$ including EWSB and phase space effects can be found on the left panel in Fig. 1.

#### LHC searches

*Single production* $T_+$ can be singly produced at the LHC in association with a third generation quark. A recent search from CMS [38] looked for $(T^+ \rightarrow Z\;t) bq$ with a fully leptonic *Z* decay. The search places a lower bound on the mass of the singlet LH Top partner at 1.2 TeV, assuming negligible width and BR$[T^+\rightarrow Z t]=0.25$. The bound strongly relies on a model-dependent production cross-section, which in term depends on the coefficients of Eq. (76). In the CMS search the coupling is fixed at $C_{bW}=0.5$. Conservatively we consider the $m_{T^+}>1.2$ TeV bound at face value, although we expect a smaller value for $C_{bW}$, as can be seen in Eq. (76). $C_{bW}$ is further suppressed for $\lambda _2>1$, which is the region in parameters space that, as we later show, is consistent with the LHC constraints on the *T*-odd top partners masses. The mass of the *T*-odd singlet is bound from below to be $m_{T^+} > \sqrt{2} f$. The lower bound of 1.2 TeV can be trivially satisfied by taking $f>850$ GeV.

*Double production* $T_+$ can also be doubly-produced via QCD processes. A recent search from ATLAS [39] looked for a pair produced top partners in a range of final states, assuming that at least one of the top partner decays to *th*. The quoted nominal bound of the singlet top partner is

80 $$\begin{aligned} m_{T^+} > 1.02~\text {TeV}. \end{aligned}$$

This bound can be satisfied by taking $f> 700$ GeV.

### *T*-odd singlet $T^-$

The phenomenology of the *T*-odd singlet resembles that of a stop squark with conserved R-parity. It can be doubly produced at the LHC via QCD processes, and consequently decay to tops and missing energy.

#### Decay modes

We consider the limit $m_{T^-} \gg m_s,m_{B_H},m_t$. $T^{-}$ couples to the singlet $\phi _0$ of the original $\frac{SU(5)}{SO(5)}$ coset. In a general $R_\xi$ gauge, $\phi _0$ is composed of the physical singlet and the would-be longitudinal component of $B_H$,

81 $$\begin{aligned} \phi _0 = s_0 s + c_0 G_0. \end{aligned}$$

The relevant interactions are82Leading to the simple prediction in the high energy limit

83 $$\begin{aligned} \Gamma (T^- \rightarrow s\; t) : \Gamma (T^- \rightarrow B_H t) = \left( \frac{s_0}{c_0}\right) ^2 = \frac{r^2}{5}. \end{aligned}$$

The exact branching ratios of $T^{-}$ including EWSB and phase space effects can be found on the right panel in Fig. 1.

#### LHC searches

We performed a simple recast of recent stop bounds by accounting for the enhanced production cross section of the fermionic $T^-$ relative to the scalar stop squark case. We would like to account for the presence of the *T*-odd doublet, which contributes to the same final states as $T^-$. We postpone the derivation of these bounds to Sect. 4.3.

### *T*-odd doublet $\psi ^-$

The phenomenology of the *T*-odd doublet resembles that of a mass-degenerate stop and sbottom squarks with conserved R-parity. The upper (lower) component up $\psi ^-$ can be doubly produced at the LHC via QCD processes, and consequently decay to tops (bottoms) and missing energy.

#### Decay modes

We consider the limit where $m_{\psi ^-} \gg m_{B_H},m_{W_H},m_s,m_\varphi$. In a general $R_\xi$ gauge, we can express our original pNGB’s in terms of the physical pNGB’s and the would-be longitudinal modes of the heavy gauge fields defined in Eqs. (51) and (53),

84a $$\begin{aligned} \begin{pmatrix} \tau ^a \\ \pi ^a \end{pmatrix} = \begin{pmatrix} c_3 &{} s_3 \\ -s_3 &{} c_3 \end{pmatrix} \begin{pmatrix} \varphi ^a \\ G^a \end{pmatrix} , \end{aligned}$$

84b $$\begin{aligned} \begin{pmatrix} \pi ^0 \\ \phi _0 \end{pmatrix} = \begin{pmatrix} c_0 &{} -s_0 \\ s_0 &{} c_0 \end{pmatrix} \begin{pmatrix} s \\ G^0 \end{pmatrix} . \end{aligned}$$

with the mixing angles $c_0,c_3,s_0,s_3$ defined in Eq. (52). The relevant interaction in the $\xi \rightarrow 0$ limit originate from $\mathcal {L}_{\kappa }$. For $(\psi ^-_R)^1$,

85 $$\begin{aligned} \mathcal {L}_{\kappa } \ni \frac{i\kappa }{2} \frac{1}{r}\Bigg [ (c_0 s-s_0 G_0) \overline{t}_L +(-s_3 \varphi _3+c_3 G_3 ) \overline{t}_L \nonumber \\ +\sqrt{2}(-s_3 \varphi ^-+c_3 G^- )\overline{b}_L\Bigg ](\psi ^-_R)^1, \end{aligned}$$

and similarly for $(\psi _-^R)^2$,

86 $$\begin{aligned} \mathcal {L}_{\kappa } \ni \frac{i\kappa }{2} \frac{1}{r} \Bigg [ (c_0 s-s_0 G_0) \overline{b}_L -(-s_3 \varphi _3+c_3 G_3 ) \overline{b}_L \nonumber \\ +\sqrt{2}(-s_3 \varphi ^++c_3 G^+ )\overline{t}_L\Bigg ](\psi ^-_R)^2. \end{aligned}$$

**Fig. 2:**
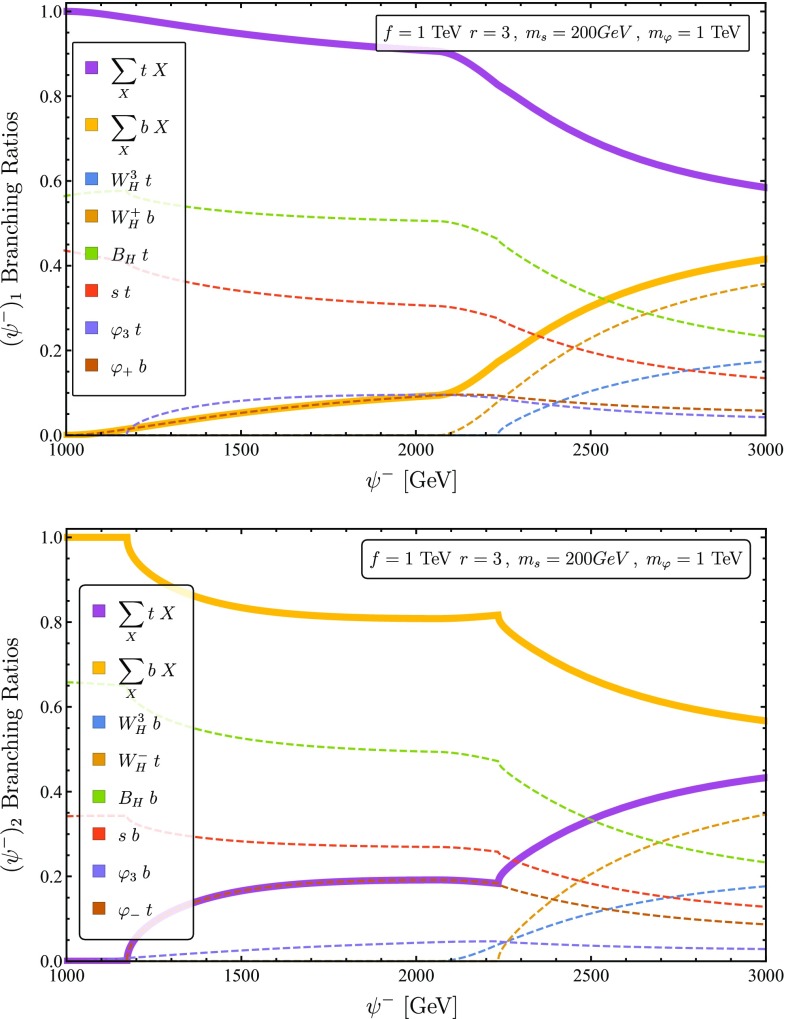
Numeric results for the branching ratios of the upper (lower) component of $\psi _-$ presented in the left (right) panel, with $f~=~1~\text {TeV},r~=~3, m_s=200~\text {GeV},m_{\varphi }=1~\text {TeV}$ and $\lambda _2=2.5$. The masses of the heavy gauge boson are fixed at $m_{B_H} = 270$ GeV and $m_{W_H} = 2.1$ TeV. The dashed colored lines indicate the branching ratios to the different exclusive final states. The solid thick lines indicate the sum of branching ratios with either a top (purple curve) or a bottom (yellow curve) at the final state

In the high energy limit

87a $$\begin{aligned} \text {Br}[\psi ^- \rightarrow q \;s]= & {} \frac{c_0^2}{4},\quad \text {Br}[\psi ^- \rightarrow q \;G_0] = \frac{s_0^2}{4},\end{aligned}$$

87b $$\begin{aligned} \text {Br}[\psi ^- \rightarrow q \;\varphi _3]= & {} \frac{1}{2} [\psi ^- \rightarrow q \;\varphi _\pm ] = \frac{s^2_3}{4},\end{aligned}$$

87c $$\begin{aligned} \text {Br}[\psi ^- \rightarrow q \;G_3]= & {} \frac{1}{2} [\psi ^- \rightarrow q \;G_\pm ] =\frac{c^2_3}{4}. \end{aligned}$$

with the final state with $q= \{b,t\}$ depending on the electric charge of the initial state. The exact branching ratios for $\psi ^{-}$ including EWSB and phase space effects can be found in Fig. 2.

**Fig. 3:**
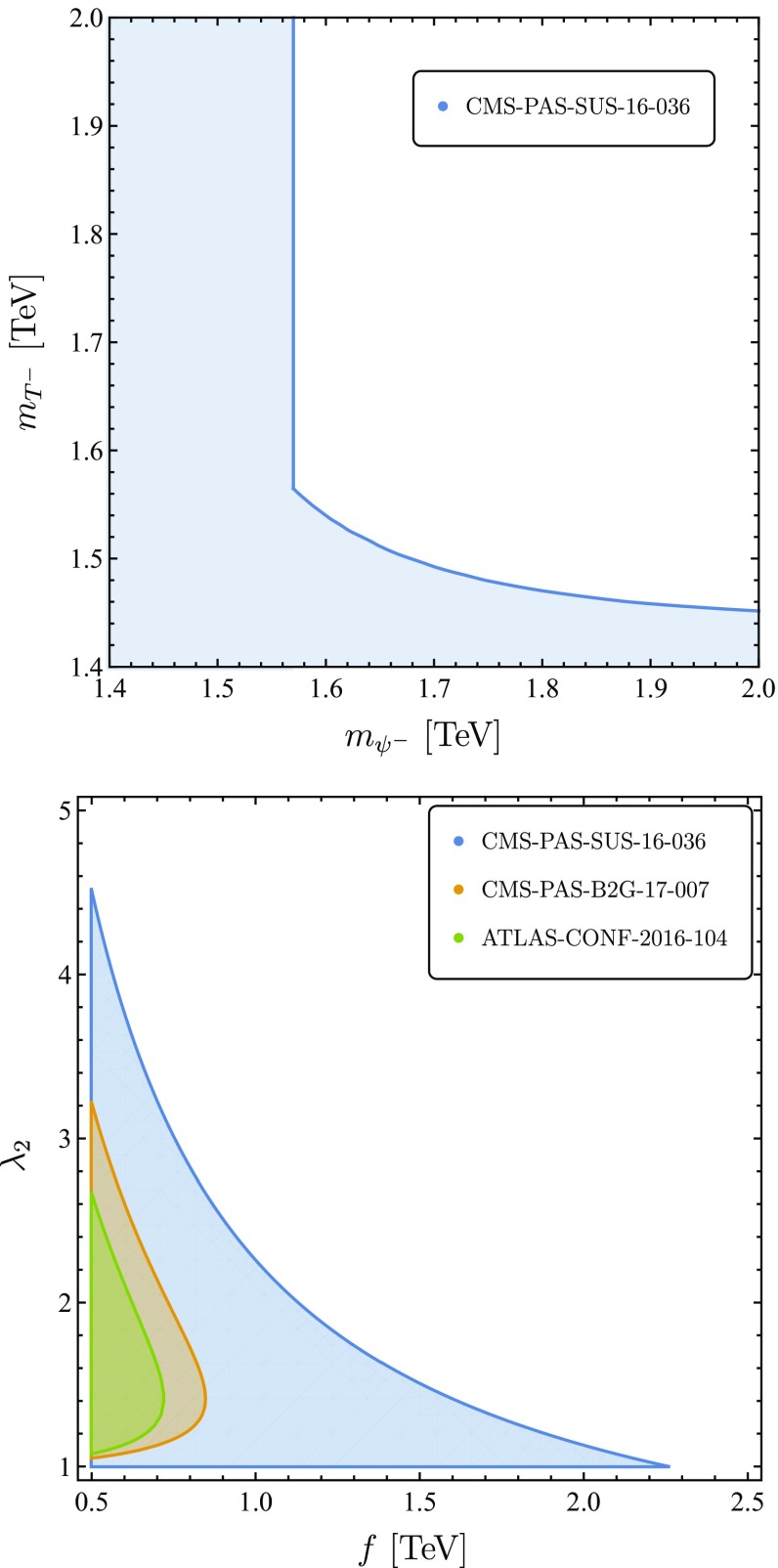
Left Panel: Exclusion limits (blue region) in the $(m_{\psi ^-},m_{T^-})$ plane, using recasted limits from the CMS SUSY search of Ref. [42]. We impose the condition of Eqs. (90) and (90), assuming branching ratios of 100%. Right Panel: Exclusion limits in the $(f,\lambda _2)$ plane using Ref. [42] (blue region, using the bound from Eq. (92)), Ref. [38] (orange region) and Ref. [39] (green region)

#### LHC searches

The *T*-odd sector contains two top-like and one bottom-like fermions. We perform a recast of recent bounds on stop and sbottom masses by accounting for the enhanced production cross section of a fermionic colored top partner, along the lines of [40] and [41]. The quoted bounds in Ref. [42] for the stop and sbottom masses are

88 $$\begin{aligned} m_{\tilde{t}} \ge 1070~\text {GeV}, \quad m_{\tilde{b}} \ge 1175~\text {GeV}, \end{aligned}$$

respectively. We denote the QCD pair production cross section at $\sqrt{s}= 13$ TeV for a spin *s* coloured particle with mass *M* as $\sigma ^s_{\text {pair}}(M)$. We require that

89 $$\begin{aligned} \sigma ^0_{\text {pair}}(1070~\text {GeV})\ge & {} \sigma ^{1/2}_{\text {pair}}\left( m_{T^-}\right) \\+ & {} \sigma ^{1/2}_{\text {pair}}(m_{\psi ^-})\times \text {BR}[(\psi _-)_1 \rightarrow t+\text {MET}] \nonumber \end{aligned}$$

and

90 $$\begin{aligned} \sigma ^0_{\text {pair}}(1175~\text {GeV})\ge & {} \sigma ^{1/2}_{\text {pair}}(m_{\psi ^-})\\&\times \text {BR}[(\psi _-)_2 \rightarrow b+\text {MET}],\nonumber \end{aligned}$$

with $m_{\psi ^-} = \kappa f$ and $m_{T^-} = \frac{\lambda _2 f}{\sqrt{2}}$ the masses of the *T*-odd doublet and *T*-odd singlet top partners respectively. We use $\sigma ^0_{\text {pair}}(M)$ reported by the CMS collaboration [43] and $\sigma ^{1/2}_{\text {pair}}(M)$ calculated using HATHOR [44]. The combination of Eqs. (90) and (90) in the $(m_{\psi ^-},m_{T^-})$ plane is plotted in the left panel of Fig. 3. We conservatively assume all the branching ratios to be $100\%$. We thus obtain the following lower bounds on the *T*-odd fermion masses

91 $$\begin{aligned} m_{\psi ^-},m_{T_-} > 1.6~\text {TeV}. \end{aligned}$$

The combination of all LHC constrains in the $(f,\lambda _2)$ plane is shown in the right panel of Fig. 3. We summarize the constraints for the couplings for a for a given *f*,

92a $$\begin{aligned}&\frac{1.6~\text {TeV}}{f}<\kappa < 4 \pi ,\end{aligned}$$

92b $$\begin{aligned}&\text {Max}\left[ 1,\frac{2.3~\text {TeV}}{f}\right]<\lambda _2 < 4 \pi . \end{aligned}$$

## Electroweak precision tests

The main contributions to electroweak precision observables are unaffected by the extended coset structure. The mixing in the left-handed sector generates a correction to the T oblique parameter due to loops of the *T*-even singlet $T^+$ [31]

93 $$\begin{aligned} T_{T^+}&= T_\text {SM} \,{s_L}^2\,\,\left[ \frac{{s_L}^2}{x_t}-2+{s_L}^2-\frac{2{s_L}^2}{1-x_t}\log x_t \right] , \end{aligned}$$

with

94a $$\begin{aligned} T_\text {SM}= & {} \frac{3}{16\pi }\,\frac{1}{s_w^2c_w^2}\,\frac{m_t^2}{m_Z^2}\approx 1.24, \end{aligned}$$

94b $$\begin{aligned} x_t\equiv & {} \frac{m_t^2}{m_{T^+}^2} \approxeq \left( \frac{\lambda _2^2-1}{\lambda _2^4}\right) \xi ,, \end{aligned}$$

and

95 $$\begin{aligned} s_L \equiv \sin \theta _L \approxeq \sqrt{\frac{x_t}{\lambda _2^2-1}} . \end{aligned}$$

We express $T_{T^+}$ in terms of $x_t$ using Eq. (95). In light of the LHC constrains on the *T*-even top partner mass of Eq. (80), we expect $x_t \le 0.03 \ll 1$. We therefore expand Eq. (93) to leading order in $x_t$:

96a $$\begin{aligned} \frac{T_{T^+}}{ T_\text {SM}}\approx & {} \left( \frac{ x_t }{\lambda _2^2-1} \right) \left( 2\log \frac{1}{x_t}+ \left[ \frac{1}{\lambda _2^2-1}\right] -2\right) \end{aligned}$$

96b $$\begin{aligned}= & {} \left( \frac{ \xi }{\lambda _2^4} \right) \left( 2\log \left[ \frac{\lambda _2^4}{(\lambda ^2_2-1)\xi }\right] + \left[ \frac{1}{\lambda _2^2-1}\right] -2\right) .\nonumber \\ \end{aligned}$$

An additional contribution to the T parameter is due to loops of *T*-odd heavy gauge bosons. The correction is proportional to the mass splitting after EWSB,

97 $$\begin{aligned} \Delta m_{W_H}^2 \equiv m_{W^3_H}^2-m_{W^\pm _H}^2 = \frac{1}{2} f^2 g^2 \sin ^4 \left( \sqrt{\frac{\xi }{2}} \right) , \end{aligned}$$

neglecting corrections of order $O(g'^2)$. The *T*-odd gauge loops generate the following correction to the T parameter [31]

98 $$\begin{aligned} T_{W_H}= & {} - \frac{9}{16 \pi c_w^2 s_w^2 M_Z^2} \Delta m_{W_H}^2 \log \left( \frac{\Lambda ^2}{f^2 g^2 (1+r^2)}\right) \nonumber \\= & {} - \frac{9}{16 \pi s_w^2 } \xi \log \left( \frac{\Lambda }{f g \sqrt{1+r^2}}\right) . \end{aligned}$$

This correction is $\lambda _2$ independent, and becomes the dominant one for higher values of $\lambda _2$ as $T_{T^+}\rightarrow 0$. We assume that the UV contributions to these loop processes are sub-leading with respect to the log-enhanced IR contribution.

Let us mention that the oblique *S* and *U* parameters also receive corrections due to the mixing the LH fermion sector. As noted in Ref. [31], the size of these corrections are an order of magnitude smaller than the correction to the *T* parameter and are therefore sub-leading. Additionally, the $Z \bar{b}_L b_L$ vertex receives corrections due to $T_+$ loops [31]

99 $$\begin{aligned} \delta g_L^{Z\bar{b}b}=\frac{g}{c_w}\frac{\alpha }{8\pi s_w^2} \frac{m_t^4}{m_W^2 m_{T^+}^2} \left( \frac{1}{\lambda _2^2-1}\right) \log \frac{m_{T^+}^2}{m_t^2}, \end{aligned}$$

with $\delta g_L^{Z\bar{b}b} \equiv g_L^{Z\bar{b}b} - g_{L\;\text {SM}}^{Z\bar{b}b}$ and $g_{L\;\text {SM}}^{Z\bar{b}b} = -\frac{1}{2} + \frac{s^2_w}{3}$. We constrain the parameters of the model using the results of Ref. [45], namely

100a $$\begin{aligned} T&= 0.12 \pm 0.07, \end{aligned}$$

100b $$\begin{aligned} \delta g_L^{Z\bar{b}b}&= 0.002 \pm 0.001. \end{aligned}$$

**Fig. 4:**
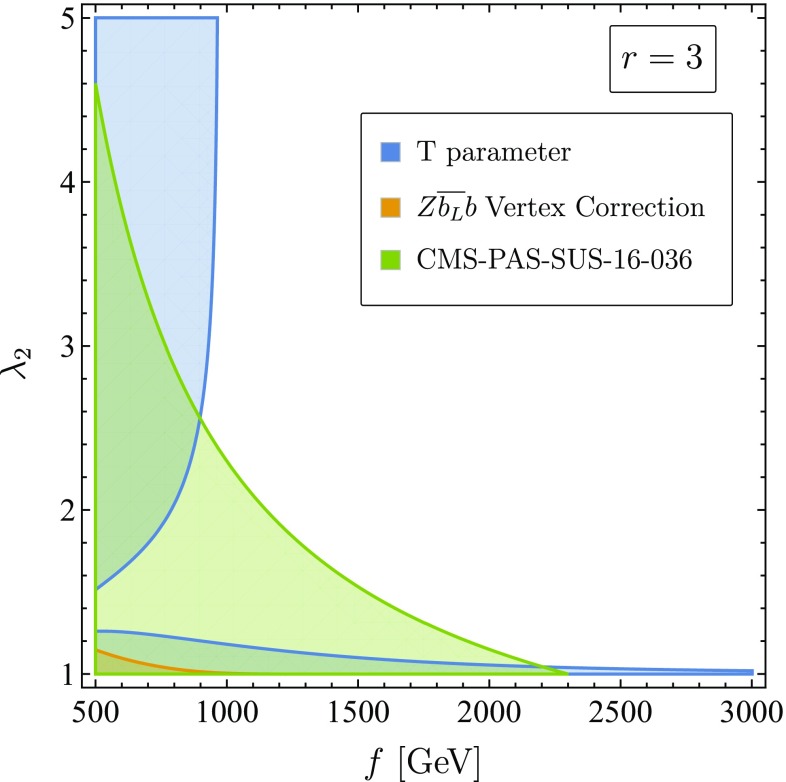
Combined EWPT and LHC exclusion regions in the $(f,\lambda _2)$ plane, for $r=3$ and $\Lambda = 4\pi f$. The EWPT exclusion regions due to T-parameter (blue region) and $\delta g_L^{Z\bar{b}b}$ (orange region) are plotted at the $3\sigma$ level using the results of Ref. [45], $T~=~0.12~\pm ~0.07$ and $\delta g_L^{b\bar{b}}~=~0.002~\pm ~0.001$. The LHC exclusion (green region) is due to Ref. [42] using the lower bound of Eq. (92)

The combinations of the EWPT and LHC constraints are plotted in  Fig. 4. For $f\lesssim 1.5$ TeV values of $\lambda _2<1.5$ are excluded by LHC. The correction $T_{T^+}$ decreases as $\lambda _2$ increases, and in the allowed regions we find that $T_{W_H} \gg T_{T^+}$. We conclude that the correction from *T*-odd gauge loops to the T-parameter is the dominant constraint in the allowed region where $\lambda _2$ is large. We find the following lower bound on *f* from Eq. (98) at $3\sigma$ after taking $\Lambda = 4 \pi f$

101 $$\begin{aligned} f> & {} (1240~\text {GeV})\times \sqrt{1-\frac{1}{6} \log (1+r^2)} \nonumber \\\approx & {} (970~\text {GeV}) ~\times ~ \left( 1-0.08(r-3))\right) . \end{aligned}$$

Therefore we set the lower bound on the symmetry breaking scale to be $f > 1~\text {TeV}$.

## Dark matter phenomenology

### Spectrum

The lightest *T*-odd particle (LTP) in the spectrum is stable and therefore a natural DM candidate. One possible LTP is the gauge field $B_H$. This possibility has been considered in the past in the context of the original LHT model [46]. In this work we explore the possibility of DM being part of the composite scalar sector, in particular the singlet *s*. The singlet mass $m_s$ is a free parameter in our model. The mass $m_{B_H}$, given in Eq. (48), is of order *O*(200) GeV. The region in which *s* is the LTP corresponds to $r \sim 2-3$ and thus would be the focus of our study. In this region we may safely neglect co-annihilation effects of *s* with $B_H$. Since larger values of *r* correspond to heavier *T*-odd gauge bosons, there is a small increase in the fine-tuning of the Higgs mass from the gauge sector. We can easily see by comparing the logarithmically divergent contributions to the Higgs mass from the two sectors

102 $$\begin{aligned} \frac{\mu ^2_{\text {gauge}}}{\mu ^2_{\text {top}}}\sim & {} \frac{g^4 (1+r^2)}{\lambda _1^2\lambda _2^2} \sim \left( \frac{g^4}{\lambda ^2_2} \right) (1+r^2) \nonumber \\\sim & {} \text {a few precent}~\times ~ (1+r^2). \end{aligned}$$

that this increase is negligible compared to the dominant source of tuning from the top sector.

### Singlet-triplet mixing

The last term of the scalar potential in Eq. (73) induces mixing between the singlet *s* and the neutral component of the triplet $\varphi _3$ after EWSB. The effects of singlet-triplet mixing on the DM phenomenology have been considered in Ref. [47]. We focus on the composite nature of the singlet DM. For simplicity, we limit ourselves to the region in parameter space where we may neglect the mixing effects. The mixing angle is given by

103a $$\begin{aligned} \sin ^2\theta _{s\varphi }= & {} \frac{1}{2}\left[ 1-\sqrt{\frac{1}{1+t^2}}\right] = \frac{t^2}{4}+O(t^4),\end{aligned}$$

103b $$\begin{aligned} t\equiv & {} \frac{1}{2}\frac{\lambda _\varphi v^2}{ |m_\varphi ^2-m_s^2|}. \end{aligned}$$

Assuming for simplicity that $m_s \sim v, \lambda _\varphi \sim 1$ and demanding conservatively that $\sin \theta _{s\varphi }~<~ 5\%$, we find the following lower bound

104 $$\begin{aligned} \frac{m_\varphi }{m_s} \gtrsim 2.5, \end{aligned}$$

which implies $m_\varphi \gtrsim 600~$GeV, consistent with current collider bounds.^1^ We note that the assumption $\lambda _\varphi \sim 1$ as well as the lower bound on $m_\varphi$ are consistent with the IR contribution of Eq. (73) to these operators. We find that the operator corresponding to $\lambda _\varphi$ enjoys an accidental factor $\sim 5$ enhancement to its coefficient in the CW potential. The IR contributions can be found in Appendix 1 in Eqs. (B.47) and (B.48). We conclude that a moderate mass separation is sufficient in order to neglect the singlet-triplet mixing effects.

### Annihilation cross section

The DM relic abundance is calculated by solving the Boltzmann equation for the particle density [50]

105 $$\begin{aligned} \dot{n}_s +3 H n_{s} = -\langle \sigma v \rangle \left[ n_s^2-(n_s^{\text {EQ}})^2\right] . \end{aligned}$$

The thermally averaged cross section for a non-relativistic gas at temperature *T* is given by [51]

106 $$\begin{aligned} \langle \sigma v \rangle = \frac{\int _{4m_s^2}^{\infty } \mathrm {d}s \; \sigma (s-4m_s^2)\sqrt{s} K_1(\sqrt{s}/T) }{8m_s^4 T K^2_2(m/T)}, \end{aligned}$$

and the usual approximation yields [50]

107 $$\begin{aligned} \frac{\Omega _s h^2}{0.12} \approx \left( \frac{3~\times ~ 10^{-26} \;\text {cm}^3\; \text {s}^{-1}}{\langle \sigma v \rangle }\right) = \left( \frac{1\; \text {pb}\; \text {c}}{\langle \sigma v \rangle }\right) . \end{aligned}$$

The measured DM relic abundance is [52]

108 $$\begin{aligned} \Omega _{\text {DM}} h^2 = 0.1199 \pm 0.0027. \end{aligned}$$

In the following we consider three types of interactions relevant to our model that determine the annihilation cross section, the Higgs portal, the derivative couplings and the contact term [5, 6].

#### Higgs portal

Due to the explicit breaking of the global symmetry, the scalar potential of Eq. (73) is generated radiatively, and in particular the following operators are present in the theory

109 $$\begin{aligned} \mathcal {L}\ni -\frac{1}{2} \tilde{m}_s^2 s^2 - \lambda _{\text {DM}}s^2 H^\dagger H. \end{aligned}$$

$\lambda _{\text {DM}}$ is the usual Higgs portal coupling of the singlet DM model [53–55]. The Higgs mediates *s*-channel annihilation to SM gauge fields and fermions. The annihilation channel $s s\rightarrow h h$ is also possible via the *s*,*t* and *u* channels as well as directly via the dimension 4 operator $s^2 h^2$. We assume that freeze-out occurs after the EW phase transition. In unitary gauge, we can rewrite Eq. (109) as

110 $$\begin{aligned} \mathcal {L}\ni -\frac{1}{2} \left( \tilde{m}_s^2+\lambda _{\text {DM}}v^2\right) s^2 - \lambda _{\text {DM}}v \; s^2 h- \frac{\lambda _{\text {DM}}}{2} s^2 h^2.\nonumber \\ \end{aligned}$$

We define the physical mass of the singlet

111 $$\begin{aligned} m_s^2 \equiv \tilde{m}_s^2+\lambda _{\text {DM}}v^2. \end{aligned}$$

As discussed in Sect. 3, we take $m_s, \lambda _{\text {DM}}$ to be free parameters. We note a posteriori that the phenomenologically viable regions not excluded by direct detection have $\lambda _{\text {DM}}\lesssim 1\%$. The naive IR contribution to $\lambda _{\text {DM}}$ is $O(10\%)$. To obtain a viable model we assume that additional contributions generate cancellations of order a few in order for the Higgs portal coupling to take smaller values. These additional contributions can originate from UV physics, e.g loops of heavier resonances, and higher loop order diagrams containing the lightest top partners . The latter can be quadratically dependent on the UV scale, since we expect the collective breaking mechanism to break down at higher loop order. These additional contributions are expected to be comparable to the leading logarithmic contributions, allowing a substantial cancelation with the leading order contributions to take place in some parts of parameter space.

#### Goldstone derivative interaction

The kinetic term of the non linear sigma model of Eq. (46) contains derivative interactions among the Goldstone fields, in particular

112 $$\begin{aligned}&\mathcal {L}_{nl\sigma } \ni \frac{5 s_0^2}{12 f^2} \left[ s (\partial _\mu s)\partial ^\mu (H^\dagger H)- s^2 (\partial ^\mu H^\dagger \partial _\mu H)\right. \nonumber \\&\qquad \qquad \left. - (\partial _\mu s )^2 H^\dagger H\right] . \end{aligned}$$

These derivative interactions scales like $m_s^2 /f^2$, and we expect them to become increasingly stronger for heavier DM masses or lower values of *f*. They affect all the annihilation channels of the Higgs portal couplings, typically resulting in destructive interferences [5]. We discuss this effect in detail in Sect. 6.4. As *r* increases, $s_0$ increases and approaches unity. This is equivalent to decreasing the effective scale of this operator $\tilde{f} = f / s_0$, thus making these interactions stronger for lower DM masses.

#### Contact term

The non-renormalizable nature of the theory and the mixing in the top sector leads to the appearance of the following contact term,

113 $$\begin{aligned} \mathcal {L}_{\text {top}}\ni \frac{c_{s^2 \bar{t} t}}{f} s^2 \bar{t}t. \end{aligned}$$

with

114 $$\begin{aligned} c_{s^2 \bar{t} t} = -y_t s_0^2 \left( \frac{2 \sqrt{2} }{5 }\right) \left( c_L\left( \frac{7 \sqrt{\xi }}{12 } \right) +s_L\right) . \end{aligned}$$

As opposed to the standard singlet DM which interacts with the SM only through the Higgs portal, this dimension 5 operator allows the singlet to annihilate directly into tops without the mediation of the Higgs. Similarly to the derivative interactions, the contact term becomes increasingly important at higher energies. At leading order in $\xi$, we obtain

115 $$\begin{aligned} c_{s^2 \bar{t} t} \approx s_0^2 \left( \frac{ 7 }{15 \sqrt{2} }\right) \sqrt{\xi }\left( 1 +\frac{12}{7 \lambda _2^2}\right) +O(\xi ). \end{aligned}$$

As *r* increases, the effective scale of this operator $\tilde{f} = f / \sqrt{s_0}$ decreases, thus making this interaction stronger for lower DM masses.

### Relic abundance

We can characterize the DM phenomenology in 3 distinct mass regions, see also [56]. In the first region where $m_s \ll \sqrt{\lambda _{\text {DM}}}f$, all the effects of the interactions originating from higher dimensional operators, namely the derivative interactions and contact term, are negligible compared to the portal coupling interaction. The DM phenomenology in this region coincides with the standard singlet DM [53–55]. In regions where $m_s \sim \sqrt{\lambda _{\text {DM}}}f$, the effect of higher dimensional operators becomes comparable with the marginal portal coupling operator. In particular we find a destructive interference between the Higgs portal coupling and the derivative interactions. Lastly, for heavy DM masses $m_s \gg \sqrt{\lambda _{\text {DM}}}f$, the higher dimensional derivative operators dominate. For the following discussion it would be useful to parameterize the thermal cross section as

116 $$\begin{aligned} \langle \sigma v \rangle= & {} \sigma _0\left( x\right) \left[ \left( \lambda _{\text {DM}}- f_1\left( x\right) \right) ^2+f_2(x)\Theta (m_s-m_t)\right] , \nonumber \\ x\equiv & {} \frac{m_s}{f}. \end{aligned}$$

$\sigma _0,f_1,f_2$ are monotonically increasing functions of *x*. Furthermore, $\sigma _0,f_1,f_2$ depend in general on $f,r,\lambda _2$. $f_1(x)$ parametrizes the destructive effects of the dimension 6 operator of Eq. (112), hence we expect $f_1 \sim x^2$ . $f_2(x)$ accounts for the dimension 5 operator of Eq. (113), which allows the singlet to annihilate into two tops independently of the Higgs interactions, therefore we expect $f_2 \sim x$.

#### Portal coupling dominance

In regions of parameter space where

117 $$\begin{aligned} m_s \ll \sqrt{\lambda _{\text {DM}}}f, \end{aligned}$$

the composite features of the DM are negligible, and the phenomenology is that of the standard singlet DM [53–55], where irrelevant operators are irrelevant. In this area of parameter space, the thermally averaged cross section is approximately

118 $$\begin{aligned} \langle \sigma v \rangle \approx \sigma _0\left( x\right) \lambda _{\text {DM}}^2, \end{aligned}$$

and the observed relic abundance is produced for

119 $$\begin{aligned} \lambda _{\text {DM}}^+(x) \approx \sqrt{\frac{1~\text {pb}}{\sigma _0\left( x\right) }}. \end{aligned}$$

For $\lambda _{\text {DM}}< \lambda _{\text {DM}}^+$ the singlet is over-abundant. These regions are experimentally excluded. In the range $\lambda _{\text {DM}}> \lambda _{\text {DM}}^+$ the singlet is under-abundant. In this region an additional source of DM must be present in order to account for the observed relic abundance. For a fixed value of *f*, this region is characterized by a large portal couplings or small DM masses. The mass region $m_s < m_h/2$ is severely constrained by the LHC due to the Higgs invisible width to singlets. For $m_s \approx m_h/2$, the Higgs mediator is resonantly produced and $\lambda _{\text {DM}}$ must be extremely suppressed in order to produce the correct relic abundance, making this finely tuned region hard to probe experimentally. We shall focus on DM masses above $m_h/2$ the avoid the above-mentioned issues.

**Fig. 5:**
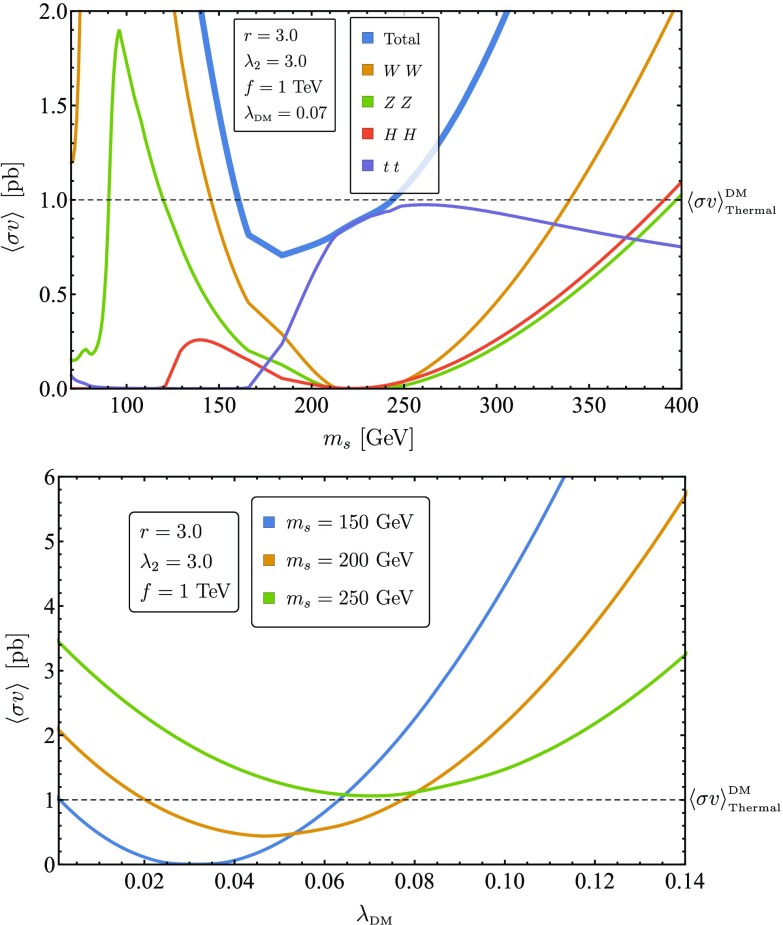
Left panel: The thermally averaged cross section as a function of the DM mass $m_s$ for $\lambda _{\text {DM}}~=~0.07,\,f~=~1000~\text {GeV},\,r=3$ and $\lambda _2=3$. The dashed line at $\langle \sigma v \rangle = 1$ pb represents the cross section that produces the correct relic abundance according to Eq. (107). Right panel: The thermally averaged cross section as a function of $\lambda _{\text {DM}}$ for different values of $m_s$ with $f=1000~\text {GeV},r=3$ and $\lambda _2=3$. The dashed line at $\langle \sigma v \rangle = 1$ pb represents the cross section that produces the correct relic abundance according to Eq. (107)

This region can be seen in the left panel of Fig. 5 where $m_s<150~$GeV. In this region the total annihilation cross section for a fixed portal coupling decreases with $m_s$, as expected in the standard singlet DM scenario for $m_s > m_h/2$. In the right panel of Fig. 5, the portal coupling dominance region is to the right of the minima of the curves. In this region, for a fixed value of the mass, the total annihilation cross section increases with $\lambda _{\text {DM}}$.

#### Contact term dominance

In region of masses where

120 $$\begin{aligned} m_s \sim \sqrt{\lambda _{\text {DM}}}f, \end{aligned}$$

the derivative interactions and Higgs portal are comparable. In this region $\lambda _{\text {DM}}\sim x^2 \sim f_1(x)$ such that the portal coupling and derivative interactions interfere destructively, implying that

121 $$\begin{aligned} \langle \sigma v \rangle \approx \sigma _0f_2(x)\Theta (m_s-m_t). \end{aligned}$$

In regions where $x < m_t/f$, $\langle \sigma v \rangle$ becomes arbitrarily small and the singlet is over-abundant. This parameter space is experimentally excluded. In the range where $x > m_t/f$ we find that $\langle \sigma v \rangle$ is positive since the singlet is kinematically allowed to decay into tops. For a particular value $x = x_{\text {max}}$ defined by

122 $$\begin{aligned} \sigma _0(x_{\text {max}})f_2(x_{\text {max}}) = 1~\text {pb}, \end{aligned}$$

the observed relic abundance is produced. In the parameter space where $m_t/f< x < x_{\text {max}}$ we find that $\langle \sigma v \rangle < 1~\text {pb}$ and the singlet is over-abundant. This range is also experimentally excluded. For coupling and masses such that $x_{\text {max}}<x$ we find that $\langle \sigma v \rangle > 1~\text {pb}$ and the singlet is under-abundant. In this region an additional source of DM must be present in order to account for the observed relic abundance. We conclude that for a given point in $(\lambda _2,r,f)$ parameter space, the largest DM mass for which the singlet can account for the entire DM relic abundance is therefore given by $m_s^{\text {max}} = \sqrt{x_{\text {max}}}f$.

The relevant parameter space in the left panel of Fig. 5 corresponds to the region where $m_s \sim 220$ GeV, close to the minimal value of the cross section. The annihilation to the Higgs and gauge bosons is effectively suppressed by the destructive interference between the portal coupling and the derivative interactions. As this suppression occurs where $m_s>m_t$, the remaining annihilation cross section is exclusively to tops. In the right panel of Fig. 5, the minima of the different curves are precisely mapped to this area of maximal interference. For the fixed mass $m_s=150$ GeV, the singlet is not allowed kinematically to decay into tops and the annihilation cross section vanishes. Conversely, for $m_s=200$ GeV the decay into tops is allowed and the annihilation cross section is dominated by the contact term. Lastly, the minimum of the curve corresponding to $m_s=250$ GeV is approximately 1 pb, meaning that for this particular point in the $(\lambda _2,r,f)$ parameter space, $x_{\text {max}} \approx 250/1000 = 1/4$.

#### Derivative interaction dominance 

In the regions of parameters space where

123 $$\begin{aligned} m_s \gg \sqrt{\lambda _{\text {DM}}}f, \end{aligned}$$

the irrelevant operators, namely the dimension 6 operators corresponding to the derivative interactions, are dominating, and the annihilation cross section grows with the singlet mass. The observed relic abundance is produced for

124 $$\begin{aligned} \lambda _{\text {DM}}^{-} \approx f_1(x)-\sqrt{\frac{1 \text { pb }}{ \sigma _0\left( x\right) }-f_2(x)\Theta (m_s-m_t)} \end{aligned}$$

for $x>x_{\text {min}}$, with $x_{\text {min}}$ defined by

125 $$\begin{aligned} f_1(x_{\text {min}})=\sqrt{\frac{1 \text { pb }}{ \sigma _0\left( x_{\text {min}}\right) }-f_2(x_{\text {min}})\Theta \left( x_{\text {min}}-\frac{m_t}{f}\right) }. \end{aligned}$$

For $x \sim x_{\text {min}}$ the correct relic abundance is recovered with $\lambda _{\text {DM}}^- \ll 1$ and with DM mass $m_s^{\text {min}} \equiv \sqrt{x_{\text {min}} }f$. The nuclear cross section is typically $\sim 10^{-11}$ pb, beyond the reach of current direct detection experiments. For $\lambda _{\text {DM}}> \lambda _{\text {DM}}^-$ the singlet is over-abundant. This regions are experimentally excluded. In the region $\lambda _{\text {DM}}< \lambda _{\text {DM}}^-$ the singlet is under-abundant. In this region an additional source of DM must be present in order to account for the observed relic abundance.

In the left panel of Fig. 5, the derivative interactions become dominant at $m_s>225$ GeV. The total annihilation cross section increases with $m_s$ for a fixed $\lambda _{\text {DM}}$, and the annihilation channels to the Higgs and gauge bosons become dominant compared to the annihilation channel to tops. In the right panel of Fig. 5 the derivative interactions dominance region can be identified to the left of the minima, where $\lambda _{\text {DM}}$ is small. The annihilation cross section increases as $\lambda _{\text {DM}}$ decreases. In this region smaller values of $\lambda _{\text {DM}}$ correspond to smaller destructive interference between the portal coupling and the derivative interactions, and therefore an increased overall annihilation cross section. For the curve corresponding to $m_s=150$ GeV, we see that $\lambda _{\text {DM}}^+ \approx 0.065$ and $\lambda _{\text {DM}}^- \ll 1$, meaning that for this particular point in the $(\lambda _2,r,f)$ parameter space, $x_{\text {min}} \approx 150/1000 = 0.15$.

**Fig. 6:**
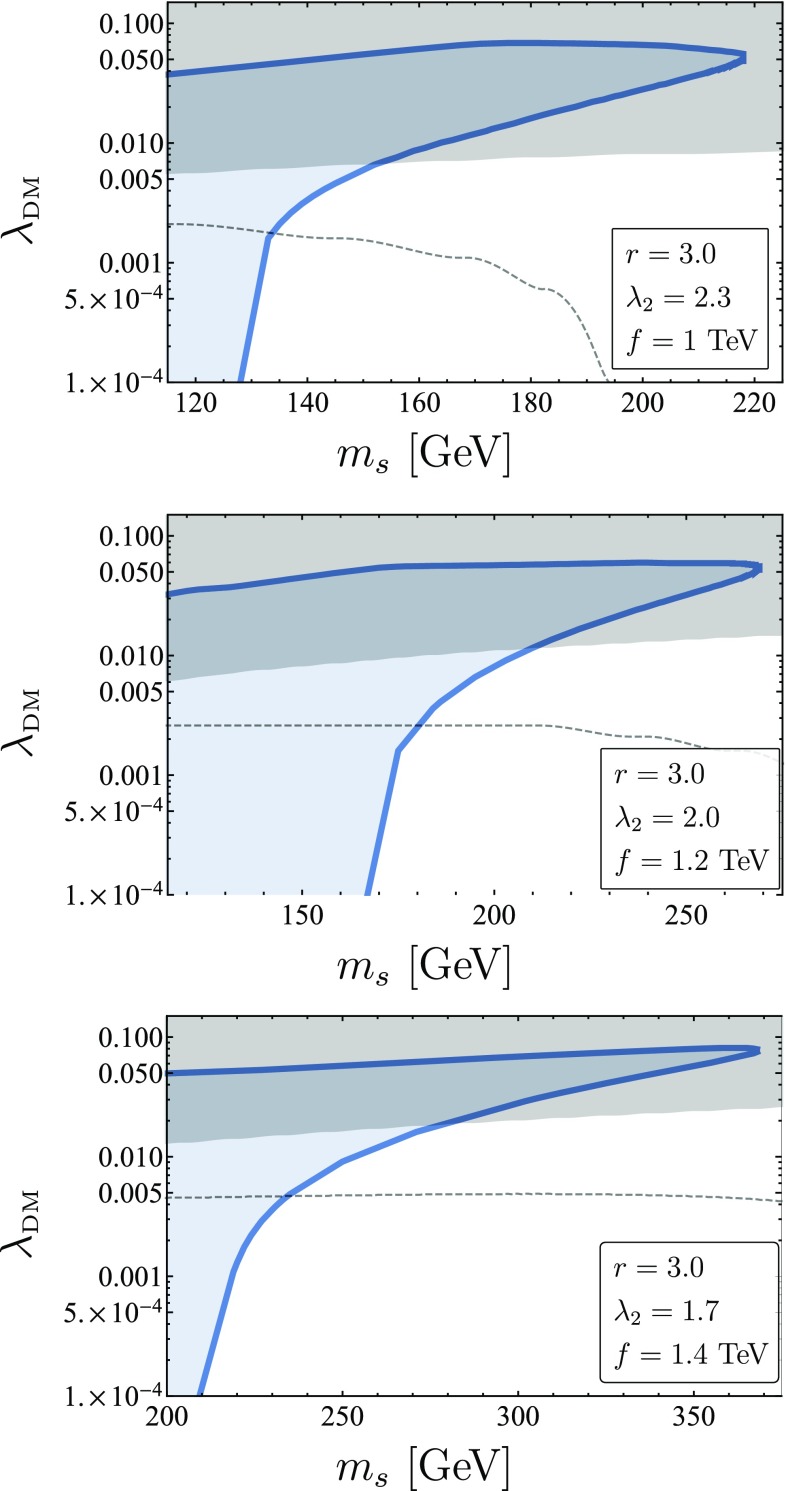
Singlet relic abundance in the $m_s,\lambda _{\text {DM}}$ plane for $f=1$ TeV (left), $f=1.2$ TeV (middle) and $f=1.4$ TeV (right), for fixed $r=3$ and minimal $\lambda _2 \sim \frac{2300~\text {GeV}}{f}$. The solid blue lines represent areas where $\Omega _s = \Omega _{\text {DM}}$. The blue areas are regions where $\Omega _s > \Omega _{\text {DM}}$, and therefore are excluded. The grey regions are excluded by XENON1T [59] after 34.2 live days. The Dashed lines are the projected sensitivities for XENON1T at $1.1 \text {yrs} \times \,\mathrm{Ton}$[60]

### Direct detection

The model was implemented using FeynRules [57] and exported to micrOMEGAs [58]. The strongest direct detection bounds are due to XENON1T [59] after 34.2 live days. Scan results for this model can be seen in Fig. 6. The two branches appearing in each panel represent the two possible solutions for $\lambda _{\text {DM}}$ for each mass value which produce the observed relic abundance. The branches meet at some maximal DM mass, above which the singlet is always under-abundant. The upper branch is ruled out by direct detection. Some of the lower branch is still consistent with experimental bounds. In the region where $m_s \approx \sqrt{x_{\text {min}}}f$, $\lambda _{\text {DM}}$ can be arbitrarily small, thus avoiding direct detection. In this regions, the theory gives a sharp prediction for the DM mass. At mentioned previously, the naive IR contribution to $\lambda _{\text {DM}}$ is too big and of $O(10\%)$. We therefore assume that additional contributions from UV physics and higher loops generate mild cancellations, allowing this coupling to take the allowed $O(1\%)$ values.

**Fig. 7:**
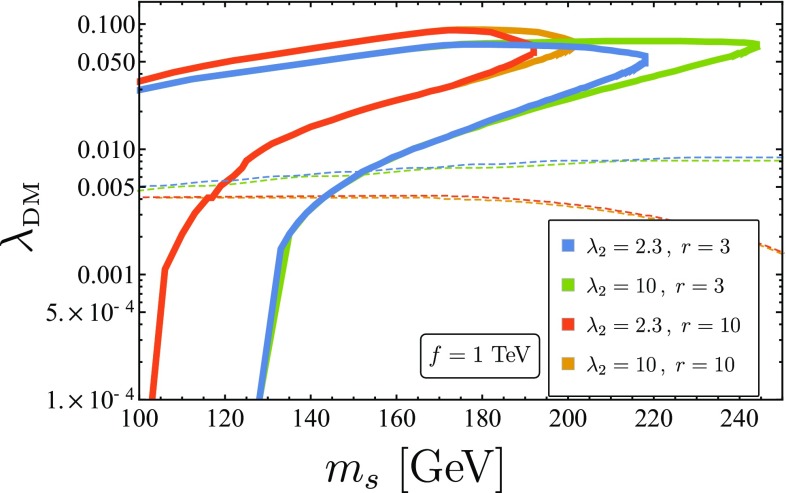
The effects of changing *r* and $\lambda _2$ on the relic abundance curves, shown as solid curves. The dashed curves represent the XENON1T [59] bounds after 34.2 live days. Increasing *r* has similar effects to lowering *f* - the coefficients of the non-renormalizable terms increase and their effect is noticeable at lower DM masses. Increasing $\lambda _2$ reduces the size of the coefficient of the dimension 5 contact term, therefore increasing $m_s^{\text {max}} = \sqrt{x_{\text {max}}}f$

The impact of varying $\lambda _2,r$ for a fixed value of *f* can be seen in Fig. 7. The largest effect is seen for increasing *r*, which in turn raises the importance of the non-renormalizable interactions at lower DM masses. A smaller effect due to the increase of $\lambda _2$ can be seen in the meeting point of the two branches. Larger values of $\lambda _2$ decrease the contact term, pushing $m_s^{\text {max}} = \sqrt{x_{\text {max}}}f$ to higher values.

## Conclusions

In this work we have presented a viable composite dark matter (DM) candidate within the Littlest Higgs with *T*-parity framework. We started by motivating a minimal extension of the original coset which allows the *T*-odd doublet to acquire a mass without introducing additional sources of explicit symmetry breaking. The extended coset contains a *T*-odd electroweak singlet. This singlet is naturally light and therefore it is reasonable to assume it is the lightest *T*-odd particle, which insures its stability.

The top sector is implemented using a collective breaking mechanism, insuring the absence of quadratically divergent contribution to the Higgs mass at 1 loop. *T*-parity implies a rich LHC phenomenology: in addition to the usual (*T*-even) top partners, the top sector contains *T*-odd top partners. This *T*-odd top partners can be doubly produced via QCD in the LHC and decay to standard model (SM) particles and missing energy. We have derived lower bounds on the masses of the *T*-even and *T*-odd top partners from various LHC searches. When combined with electroweak-precision-test (EWPT) bounds, we derived a set of constraints on the parameter space of the model.

We examined the DM phenomenology of the composite singlet DM within the allowed parameter space. The usual “elementary” singlet DM scenario is heavily constrained by direct detection experiments. In the composite singlet DM scenario, the composite nature of the DM allows it to escape detection in areas with $O(1\%)$ portal coupling, while still producing the observed relic abundance via its derivative interactions with the Higgs. The “elementary” singlet can only hide in the finely tuned “resonance” valley where $m_s \approx m_h/2$. Conversely, the composite singlet can exist in a broader region, corresponding to different values of *f* and *r*, in which it can evade detection. In these regions the correct relic abundance can be produced only due to the derivative interactions. The small portal coupling needed in these regions would in general require some mild amount of fine tuning, unless one can find a way to suppress it e.g using symmetries or additional dynamics.

## Appendix A:The complete Lagrangian

The model is defined by the global symmetry

A.1 $$\begin{aligned} \mathcal {G}= & {} SU(5) \\ \nonumber\times & {} [SU(2)_L \times U(1)_L] \times [ SU(2)_R \times U(1)_R] \times U(1)_Q. \end{aligned}$$

A global unbroken $U(1)_Q$ is added in order to fix the hyper charges of the matter fields. *SU*(5) contains two $SU(2)~\times ~ U(1)$ subgroups defined in Eqs. (5a) and (5b), denoted by $[SU(2)~\times ~ U(1)]_{1/2}$. We gauge the following subgroup

A.2 $$\begin{aligned} SU(2)_{1+L}\times U(1)_{1+L+Q} \times SU(2)_{2+R} \times U(1)_{2+R+Q}.\nonumber \\ \end{aligned}$$

We implicitly include $SU(3)_c$ as an external gauge symmetry. We introduce fields in representations of $\mathcal {G}$ denoted by $(\mathcal {R},\mathcal {R}_L,\mathcal {R}_R)_{q_L,q_R,q_Q}$. A generic representation of Eq. (A.1) is mapped under *T*-parity to

A.3 $$\begin{aligned} (\mathcal {R},\mathcal {R}_L,\mathcal {R}_R)_{q_L,q_R,q_Q} \rightarrow (\overline{\mathcal {R}},\mathcal {R}_R,\mathcal {R}_L)_{q_R,q_L,q_Q}. \end{aligned}$$

The representation $\overline{\mathcal {R}}$ is defined by the automorphism of Eq. (9). The Lagrangian is described by the following sum

A.4 $$\begin{aligned} \mathcal {L}= \mathcal {L}_{\text {gauge}} + \mathcal {L}_{\text {kin}} + \mathcal {L}_{\text {top}}+ \mathcal {L}_{\kappa }. \end{aligned}$$

The gauge kinetic terms are given as usual by

A.5 $$\begin{aligned} \mathcal {L}_{\text {gauge}} =- & {} \frac{1}{4} \sum _{i=1,2}W_{ia}^{\mu \nu } W^{ia}_{\mu \nu } -\frac{1}{4} \sum _{i=1,2}B_i^{\mu \nu } B^i_{\mu \nu } \nonumber \\- & {} \frac{1}{4} G_a^{\mu \nu } G^a_{\mu \nu }. \end{aligned}$$

We introduce two scalar fields with the following $\mathcal {G}$ representation

A.6 $$\begin{aligned} \Sigma : ( {\mathbf {15}} ,{\mathbf {1}},{\mathbf {1}})_{0,0,0}, \quad X : ( {\mathbf {1}} ,{\mathbf {2}},\overline{{\mathbf {2}}})_{q_X,-q_X,0}. \end{aligned}$$

The charges of *X* under $U(1)_L ~\times ~ U(1)_R$ are constrained by the requirement to preserve the *T*-even combination $U(1)_{R+L}$. We determine the value of $q_X$ in Eq. (A.21). The global symmetry is spontaneously broken by the VEV’s of $\Sigma$ and *X* with the following coset structure

A.7 $$\begin{aligned} \frac{SU(5) }{SO(5) }\times \frac{ [ SU(2) \times U(1) ]_{L}\times [ SU(2) \times U(1) ]_{R} \times U(1)_Q}{ [ SU(2) \times U(1) ]_{L+R}\times U(1)_Q}. \nonumber \\ \end{aligned}$$

We parametrize $\Sigma$ and *X* following Eqs. (7) and (40) and write down the kinetic terms of the non-linear sigma model

A.8 $$\begin{aligned} \mathcal {L}_{\text {nl}\sigma }= & {} \frac{f^2}{8}\text {Tr}[(D_\mu \Sigma ) (D^\mu \Sigma ^*)] + \frac{{f'}^2}{4}\text {Tr}[(D_\mu X) (D^\mu X^\dagger ) ],\nonumber \\ \end{aligned}$$

with

A.9 $$\begin{aligned} D \Sigma= & {} \partial \Sigma -i \sum _{i=1,2} g_i W^a_i ( Q_i^a \Sigma + \Sigma {Q_i^a}^T) \nonumber \\&-i \sum _{i=1,2} g'_i B_i ( Y_i \Sigma + \Sigma {Y_i}^T), \end{aligned}$$

A.10 $$\begin{aligned} D X= & {} \partial X - \frac{i}{2} (g_1 W_1^a \sigma ^a X - g_2 W_2^a X\sigma ^a) \nonumber \\&- i q_X (g'_1 B_1-g'_2 B_2). \end{aligned}$$

*T*-parity dictates that

A.11 $$\begin{aligned} g_1=g_2=\sqrt{2}g, \quad g'_1=g'_2=\sqrt{2}g', \end{aligned}$$

with $g,g'$ the SM gauge couplings.

The matter sector contains the following linearly transforming fields

A.12a $$\begin{aligned} \Psi _1= & {} \begin{pmatrix} \psi _1 \\ \chi _1 \\ 0 \end{pmatrix} : (\overline{\mathbf {5}},{\mathbf {1}},{\mathbf {1}})_{0,0,\frac{1}{3}}, \end{aligned}$$

A.12b $$\begin{aligned} \Psi _2= & {} \begin{pmatrix} 0 \\ \chi _2 \\ \psi _2 \end{pmatrix} : ({\mathbf {5}},{\mathbf {1}},{\mathbf {1}})_{0,0,\frac{1}{3}}, \end{aligned}$$

and

A.13a $$\begin{aligned} \tau _1&:&({\mathbf {1}},{\mathbf {1}},{\mathbf {1}})_{\frac{8}{15},\frac{2}{15},0} ,\end{aligned}$$

A.13b $$\begin{aligned} \tau _2&:&({\mathbf {1}},{\mathbf {1}},{\mathbf {1}})_{\frac{2}{15},\frac{8}{15},0} ,\end{aligned}$$

A.13c $$\begin{aligned} \tilde{t}_R&:&({\mathbf {1}},{\mathbf {1}},{\mathbf {1}})_{0,0,\frac{1}{3}}. \end{aligned}$$

We introduce a non linearly transforming doublet $\psi ^-_R$. Non-linear representations are described in terms of representations of the unbroken subgroup

A.14 $$\begin{aligned} \mathcal {H} = SO(5) \times SU(2)_{L+R} \times U(1)_{L+R}\times U(1)_Q. \end{aligned}$$

$\psi ^-_R$ transforms non-linearly under the full global group $\mathcal {G}$ using the CCWZ formalism. In our case

A.15 $$\begin{aligned} \psi ^-_R : ({\mathbf {1}},{\mathbf {2}})_{q_X,q_\psi } \quad \text {under} \quad \mathcal {H}. \end{aligned}$$

The $U(1)_Q$ charge of $\psi ^-_R$ , denoted here by $q_{ \psi }$, is determined in Eq. (A.21). $q_X$ is the same charge appearing in Eq. (A.10). Under *T*-parity,

A.16a $$\begin{aligned}&\Psi _1 \rightarrow \Omega \Sigma _0 \Psi _2,\quad \tau _1 \leftrightarrow \tau _2, \end{aligned}$$

A.16b $$\begin{aligned}&\tilde{t}_R \rightarrow \tilde{t}_R,\quad \psi ^-_R \rightarrow -\psi ^-_R. \end{aligned}$$

The *U*(1) charge assignments are fixed by matching the required SM hyper charges and requiring that all the gauged *U*(1) symmetries are conserved. The SM hyper charge is given by

A.17 $$\begin{aligned} Y_{\text {SM}} = Y_1+Y_2+q_L+q_R+2q_Q. \end{aligned}$$

e.g for $$\psi _1$$,

A.18 $$\begin{aligned} Y_{\text {SM}} = -\frac{3}{10}-\frac{2}{10}+0+0+2\times \frac{1}{3} = \frac{1}{6}. \end{aligned}$$

Let us determine the $q_X$ and $q_\psi$ charges.

Defining $U \equiv e^{\frac{i \Pi _X}{f'}}$, the combinations $U \psi ^-_R$ and $U^\dagger \psi ^-_R$ transform linearly under the global group

A.19 $$\begin{aligned} U \psi ^-_R : ({\mathbf {1}},{\mathbf {2}},{\mathbf {1}})_{q_X,0,q_{\psi }}, \quad U^\dagger \psi ^-_R : ({\mathbf {1}},{\mathbf {1}},{\mathbf {2}})_{0,q_X,q_{\psi }}. \end{aligned}$$

Using these identifications as linear representations, it is clear that conservation of $U(1)_{1+L+Q}$ and $U(1)_{2+R+Q}$, e.g in the first term of Eq. (70), requires

A.20 $$\begin{aligned} - \left( -\frac{3}{10}+\frac{1}{3} \right) + q_X + q_\psi = 0 \end{aligned}$$

and

A.21 $$\begin{aligned} - \left( -\frac{1}{5}+\frac{1}{3} \right) + q_\psi = 0 \rightarrow q_\psi = \frac{2}{15}, \; q_X = -\frac{1}{10} . \nonumber \\ \end{aligned}$$

We introduce the kinetic terms

A.22

The kinetic term for the non-linearly transforming doublet $\psi ^-_R$ is given by

A.23 $$\begin{aligned} D_\mu \psi ^-_R = (\partial _\mu + e_\mu -iq_{ \psi } (g'_1B_{1\mu }+ g'_2B_{2\mu })) \psi ^-_R. \end{aligned}$$

The $e_\mu \equiv e^i_\mu T^i$ symbol of the CCWZ formalism connects the non-linearly transforming field and the NGB’s via the matrix *U* [34, 35]

A.24a $$\begin{aligned}&U^\dagger (D_\mu U) \equiv d^j_\mu X^j + e^i_\mu T^i, \end{aligned}$$

A.24b $$\begin{aligned}&D_\mu U = \left( \partial _\mu -ig_1 W^a_1 \frac{\sigma ^a}{2}-i q_X g'_1 B_1\right) . \end{aligned}$$

Using the automorphism defined by *T*-parity we can also write

A.25a $$\begin{aligned}&U (D_\mu U^\dagger ) \equiv -d^j_\mu X^j + e^i_\mu T^i, \end{aligned}$$

A.25b $$\begin{aligned}&D_\mu U^\dagger = \left( \partial _\mu -ig_2 W^a_2 \frac{\sigma ^a}{2}-i q_X g'_2 B_2\right) . \end{aligned}$$

This automorphism allows us to write the $e_\mu$ symbol in terms of the pion matrix *U* and the gauge fields

A.26 $$\begin{aligned} e_\mu = \frac{1}{2} \left( U^\dagger D_\mu U+U D_\mu U^\dagger \right) . \end{aligned}$$

The covariant derivatives of $\Psi _1$ and $\Psi _2$ are

A.27 $$\begin{aligned} D_\mu \Psi _1= & {} \Bigg (\partial _\mu + i \sum _{i=1,2}[g_i W_{i\mu }^a Q^{a*}_i+g'_i B_{i\mu } Y^*_i] \nonumber \\&- \frac{i}{3} (g'_1B_{1\mu }+ g'_2B_{2\mu })\Bigg ) \Psi _1,\end{aligned}$$

A.28 $$\begin{aligned} D_\mu \Psi _2= & {} \Bigg (\partial _\mu -i \sum _{i=1,2}[g_i W_{i\mu }^a Q^a_i+g'_i B_{i\mu } Y_i]\nonumber \\&- \frac{i}{3} (g'_1B_{1\mu }+ g'_2B_{2\mu })\Bigg ) \Psi _2. \end{aligned}$$

The covariant derivative of a singlet field $\chi$ transforming as $({\mathbf {1}},{\mathbf {1}},{\mathbf {1}})_{q_L,q_R,q_Q}$ is given by

A.29 $$\begin{aligned} D_\mu \chi = (\partial _\mu - i (q_L+q_Q) g'_1B_{1\mu } - i (q_R+q_Q) g'_2B_{2\mu } ) \chi .\nonumber \\ \end{aligned}$$

For completeness we report the top sector Lagrangian

A.30a $$\begin{aligned} \mathcal {L}_{\text {top}}= & {} \frac{\lambda _1 f}{2}\left( {\overline{\Psi }_1}_i O_i + (\overline{\Psi }_2 \Omega \Sigma _0)_i \tilde{O}_i \right) \tilde{t}_R \nonumber \\&+\frac{\lambda _2 f}{\sqrt{2}}\left( \overline{\chi }_1 \tau _1 - \overline{\chi }_2 \tau _2 \right) +\text {h.c}, \end{aligned}$$

A.30b $$\begin{aligned} O_i\equiv & {} \epsilon _{ijk}\Sigma _{j4}\Sigma _{k5},\quad \tilde{O}_i \equiv 2 \epsilon _{ijk}\tilde{\Sigma }_{j4}\tilde{\Sigma }_{k5}, \end{aligned}$$

and the terms that gives the *T*-odd doublet combination a mass

A.31 $$\begin{aligned} \mathcal {L}_{\kappa } = \frac{\kappa f}{\sqrt{2}}\left( \overline{\psi }_1 \sigma _2 U - \overline{\psi }_2 \sigma _2 U^\dagger \right) \psi _R^- +\text {h.c}. \end{aligned}$$

## Appendix B:The scalar potential and its symmetries

In this appendix we discuss in detail the symmetry structure of the model and the scalar potential. The Higgs doublet is protected by two different shift symmetries. Each of the shift symmetries is contained inside a different *SU*(3) subgroup of *SU*(5)

B.32a $$\begin{aligned} \exp \left[ \frac{i}{\sqrt{2} f}\begin{pmatrix} &{}\mathbf {\epsilon }&{}\phantom {10}\\ \mathbf {\epsilon }^T&{}&{}\\ &{}&{} \end{pmatrix}\right] \in [SU(3)]_1, \end{aligned}$$

B.32b $$\begin{aligned} \exp \left[ \frac{i}{\sqrt{2} f}\begin{pmatrix} \phantom {10}&{}&{}\\ &{}&{}\mathbf {\epsilon }^T\\ &{}\mathbf {\epsilon }&{} \end{pmatrix} \right] \in [SU(3)]_2. \end{aligned}$$

All the couplings that explicitly break the global symmetry in this model, namely the gauge couplings and the top sector couplings, preserve at least one of the *SU*(3) subgroups. A Higgs potential is generated only when at least two couplings are non zero, such that all the shift symmetries are broken. This so-called “Collective Breaking” mechanism insures the absence of quadratically divergent contributions to the Higgs mass. The couplings and their *T*-parity conjugate respect different symmetries, therefore it is useful to denote the T-conjugate couplings with a tilde

B.33 $$\begin{aligned} \mathcal {L}_{\text {top}}= & {} \frac{ f}{4}\left( \lambda _1 {\overline{\Psi }_1}_i O_i+ \tilde{\lambda }_1 (\overline{\Psi }_2 \Omega \Sigma _0)_i \tilde{O}_i \right) \tilde{t}_R \nonumber \\&+\frac{ f}{\sqrt{2}}\left( \lambda _2\overline{\chi }_1 \tau _1 - \tilde{\lambda }_2\overline{\chi }_2 \tau _2 \right) +\text {h.c}, \end{aligned}$$

B.34 $$\begin{aligned} \mathcal {L}_{\kappa }= & {} \frac{ f}{\sqrt{2}}\left( \kappa \overline{\psi }_1 \sigma _2 U -\tilde{\kappa }\overline{\psi }_2 \sigma _2 U^\dagger \right) \psi _R^- +\text {h.c}. \end{aligned}$$

In order to better understand the structure of the generated scalar potential, we assign spurionic *U*(1) charges to our fields and couplings, which can be found in Table 1.

**Table 1:** Spurionic *U*(1) assignment for the couplings and fields

$\Psi _1$	$\Psi _2$	*t*	$\tau _1$	$\tau _2$	$\psi _{R}^-$
*a*	*b*	*c*	*d*	*e*	*f*
$\lambda _1$	$\tilde{\lambda }_1$	$\lambda _2$	$\tilde{\lambda }_2$	$\kappa$	$\tilde{\kappa }$
$a-c$	$b-c$	$a-d$	$b-e$	$a-f$	$b-f$

The combinations of couplings appearing in the quadratically divergent contribution to the scalar potential must be of the form $g g^\dagger$ or $\tilde{g} \tilde{g}^\dagger$. We can deduce from the residual symmetries a generic form for the quadratically divergent potential. For concreteness let us consider the coupling $\lambda _1$ and set all the other explicit symmetry breaking couplings to zero. The original coset in Eq. (A.7) contains $(24-10)+ (9-5) = 18$ NGB’s, out of which 4 are eaten, leaving us with 14 physical NGB’s with the following $SU_L(2)\times U_Y(1)$ representations

B.35 $$\begin{aligned} \mathbf {3}_{\pm 1} \oplus \mathbf {2}_{\pm 1/2} \oplus \mathbf {3}_0 \oplus \mathbf {1}_0. \end{aligned}$$

Turning on only $\lambda _1$ breaks the global symmetry and changes the coset structure

B.36 $$\begin{aligned}&\frac{SU(3)\times [ SU(2) \times U(1) ]_{2}}{ [ SU(2) \times U(1) ]_{1+2}}\nonumber \\&\quad \times \frac{ [ SU(2) \times U(1) ]_{L}\times [ SU(2) \times U(1) ]_{R} \times U(1)_Q}{ [ SU(2) \times U(1) ]_{L+R}\times U(1)_Q} ,\nonumber \\ \end{aligned}$$

This coset contains $(8+4)-4 + (9-5) = 12$ NGB’s, out of which 4 are eaten, leaving us with 8 physical NGB’s with the following $SU_L(2)~\times ~ U_Y(1)$ representations

B.37 $$\begin{aligned} \mathbf {2}_{\pm 1/2} \oplus \mathbf {3}_0 \oplus \mathbf {1}_0. \end{aligned}$$

There must exist a non-linear combination of the goldstone fields

B.38 $$\begin{aligned} \tilde{\Phi }_{ij}\equiv & {} f_1\left( 1,\frac{s}{f},\frac{s^2}{f^2},\frac{\varphi ^2}{f^2},\frac{|H|^2}{f^2},...\right) \Phi _{ij} \nonumber \\&+ f_2\left( 1,\frac{s}{f},\frac{s^2}{f^2},\frac{\varphi ^2}{f^2},\frac{|H|^2}{f^2},...\right) \frac{H_i H_j }{f} \end{aligned}$$

with $f_1,f_2$ some functions of gauge-invariants, such that the quadratically divergent potential can be written as gauge-invariant function of only $\tilde{\Phi }$

B.39 $$\begin{aligned} V(\Phi ,H,s,\varphi ) = V(\tilde{\Phi }) . \end{aligned}$$

This constraint limits the form of the quadratically divergent scalar potential. E.g the mass term in the RHS of Eq. (B.39) would appear in the original NGB basis as

B.40 $$\begin{aligned}&\Lambda ^2|\lambda _1|^2\text {Tr}[\tilde{\Phi }\tilde{\Phi }^*] = \nonumber \\&\Lambda ^2|\lambda _1|^2(|f_1|^2 \text {Tr}[\Phi \Phi ^*]+\frac{1}{f} f_1 f_2^* \text {Tr}[H^\dagger \Phi H^*]\nonumber \\&\quad +\frac{1}{f}f^*_1 f_2 \text {Tr}[H^T \Phi ^* H]+\frac{1}{f^2}|f_2|^2 (H^\dagger H)^2). \end{aligned}$$

This argument can be repeated for every coupling $c \in \{ \tilde{\lambda }_1,g_1,g_2,g'_1,g'_2\}$ which generates a scalar potential proportional to $|c|^2 \Lambda ^2$. We can immediately see that the symmetry structure allows a quadratically divergent mass term for $\Phi$. The collective breaking structure prevents the appearance of $|H|^2$ in the quadratically divergent potential, as well other operators, such as

B.41 $$\begin{aligned} s^2, \varphi ^2, s^2 |H|^2\quad \text {and} \quad s H^\dagger \varphi H. \end{aligned}$$

Logarithmically divergent 1-loop contributions to the scalar potential contain four couplings. Possible combinations are trivial combination like $|c|^2 |c'|^2$, and non trivial combinations like

B.42 $$\begin{aligned} \lambda _1 \tilde{\lambda }_1^\dagger \tilde{\kappa }\kappa ^\dagger ,\,\, \tilde{\lambda }_1 \lambda _1^\dagger \kappa \tilde{\kappa }^\dagger + \text {T-conjugates}. \end{aligned}$$

At this level all scalar operators can be generated except the singlet mass. The singlet remains exactly massless at 1-loop and must acquire a mass from higher order loops, e.g 2-loop diagram by closing the Higgs loop in the 1-loop induced $s^2 |H|^2$ interaction. We report the radiatively generated couplings calculated from Eq. (72) after setting all the T-conjugate couplings to their respective values $\tilde{c} =c$. We neglect the gauge contributions which generate $O(1\%)$ corrections to the fermion loops contribution. We define $C \equiv \frac{N_c}{16 \pi ^2}a_2 \log \left( \frac{\Lambda ^2 }{f^2 }\right)$. Note that $C \sim 0.1$ for $a_2=1$ and $\Lambda = 4 \pi f$.

B.43 $$\begin{aligned}&m^2_\Phi = \frac{ N_c}{4 \pi ^2}a_1 |\lambda _1|^2 \Lambda ^2, \end{aligned}$$

B.44 $$\begin{aligned}&\lambda = \frac{ N_c}{16 \pi ^2}a_1 |\lambda _1|^2 \left( \frac{\Lambda }{f}\right) ^2, \end{aligned}$$

B.45 $$\begin{aligned}&\lambda _{\text {DM}} = \frac{C|\lambda _1|^2 r^2 \left( 25 |\lambda _2|^2 +6 |\kappa |^2 (r+5)^2\right) }{30 \left( r^2+5\right) } > 2.3 C, \nonumber \\\end{aligned}$$

B.46 $$\begin{aligned}&\mu ^2 = -C f^2 |\lambda _1|^2 |\lambda _2|^2 < -4C f^2, \end{aligned}$$

B.47 $$\begin{aligned}&\lambda _{\varphi } = \frac{C |\lambda _1|^2 r^2 \left( 5 |\lambda _2|^2+6 \kappa ^2 (r+1) (r+5)\right) }{3 \sqrt{5} \sqrt{r^4+6 r^2+5}} > 5.2C, \nonumber \\ \end{aligned}$$

The bounds are calculated assuming $\kappa ,r>1$. We find the minimal/maximal value with respect to $\lambda _1,\lambda _2$ under the top Yukawa constraint. The triplet mass is generated only from gauge loops.

We define $D\equiv \frac{3}{64 \pi ^2}a_4 \log \left( \frac{\Lambda ^2 }{f^2}\right) = \frac{C}{4} \frac{a_4}{a_2}\sim 0.025$. The triplet mass is given by

B.48 $$\begin{aligned} m^2_\varphi= & {} D f^2\left( \frac{8g^4r^2 (1 + r)^2}{1 + r^2}\right) \nonumber \\\approx & {} \left( \frac{D}{0.025} \right) \left( \frac{f}{1200~\text {GeV}}\right) ^2 \left( 850~\text {GeV}\right) ^2 , \end{aligned}$$

where we used $r=3$. Lastly, we report the Higgs potential. The Higgs potential in the unitary gauge up to order $O(\sin ^4 h)$

B.49 $$\begin{aligned} V_{h}= & {} \frac{N_c}{16 \pi ^2}f^2 \Lambda ^2a_1 \left( |\lambda _1|^2 +|\tilde{\lambda }_1|^2 \right) s_h^4\nonumber \\&+Cf^4\left( |\tilde{\lambda }_1|^2|\tilde{\kappa }|^2 -\tilde{\lambda }_1 \lambda _1^\dagger \kappa \tilde{\kappa }^\dagger -|\tilde{\lambda }_1|^2|\tilde{\lambda }_2|^2 +[g \leftrightarrow \tilde{g}] \right) s_h^2 \nonumber \\&+\frac{1}{4}Cf^4 \left( -|\lambda _1|^2| \tilde{\lambda }_1|^2-|\lambda _1|^2|\lambda _1|^2+2 |\tilde{\lambda }_1|^2 |\tilde{\lambda }_2 |^2 \right. \nonumber \\&\left. +4 \left( \lambda _1^\dagger \tilde{\lambda }_1 \kappa \tilde{\kappa }^\dagger - |\tilde{\lambda }_1|^2 |\tilde{\kappa }|^2 \right) +[g \leftrightarrow \tilde{g}]\right) s_h^4\left( \frac{h}{\sqrt{2} f}\right) . \nonumber \\ \end{aligned}$$

where we defined $s_h^n \equiv \sin ^n \left( \frac{h}{\sqrt{2} f} \right)$. After setting $\tilde{g} = g$, we find

B.50 $$\begin{aligned} V_{h}= & {} -2Cf^4|\lambda _1|^2|\lambda _2|^2s_h^2\nonumber \\&+ \left[ \frac{N_c}{8 \pi ^2} \Lambda ^2a_1 +Cf^2\left( |\lambda _2|^2-|\lambda _1|^2 \right) \right] f^2 |\lambda _1|^2 s_h^4.\nonumber \\ \end{aligned}$$

Although terms proportional to $|\lambda _1|^2 |\kappa |^2$ could have appeared a priori in the Higgs potential, they vanish due to *T*-parity. Clearly if we were to set $\tilde{\kappa }= -\kappa$, which is equivalent to flipping the parity of $\psi ^-_R$ and coupling it to the *T*-even combination $\frac{1}{\sqrt{2}}(\psi _1+\psi _2)$, the $\kappa$ coupling would have appeared in the Higgs potential. Since $\kappa$ does not appear in the Higgs potential, taking large values of $\kappa$ would have no influence on the tuning of the Higgs potential at one loop.

## Footnotes

1 The LHC phenomenology of $\varphi$ resembles that of the Wino, which implies that the charged components can be doubly produced via electroweak processes and decay to $W^\pm$ and missing energy. However, the relevant SUSY searches, e.g [48, 49], do not pose strong constraints on $m_{\varphi }$, especially in light of the reduced production cross section of the scalar triplet in comparison to the fermionic Wino.
